# Isopentenyl diphosphate isomerase exerts limited control over terpenoid biosynthesis in two woody plant species

**DOI:** 10.1093/plphys/kiag225

**Published:** 2026-04-21

**Authors:** Toni Krause, Kristina Kshatriya, Jia Zhang, Johann M Rohwer, Jonathan Gershenzon, Axel Schmidt

**Affiliations:** Department of Biochemistry, Max-Planck-Institute for Chemical Ecology, Hans-Knoell-Str. 8, Jena 07745, Germany; Department of Biochemistry, Max-Planck-Institute for Chemical Ecology, Hans-Knoell-Str. 8, Jena 07745, Germany; Department of Biochemistry, Max-Planck-Institute for Chemical Ecology, Hans-Knoell-Str. 8, Jena 07745, Germany; Laboratory for Molecular Systems Biology, Department of Biochemistry, Stellenbosch University, Private Bag X1, Matieland, Stellenbosch 7602, South Africa; Department of Biochemistry, Max-Planck-Institute for Chemical Ecology, Hans-Knoell-Str. 8, Jena 07745, Germany; Department of Biochemistry, Max-Planck-Institute for Chemical Ecology, Hans-Knoell-Str. 8, Jena 07745, Germany

## Abstract

Terpenoid biosynthesis involves linear prenyl diphosphate intermediates of various chain lengths. These are constructed from 2 C_5_ precursors, the starter unit dimethylallyl diphosphate (DMADP) and the extender unit, isopentenyl diphosphate (IDP). Isopentenyl diphosphate isomerase (IDI) alters the DMADP:IDP ratio and may furnish a specific blend of C_5_ precursors appropriate for the length of intermediates being formed in each cellular compartment. We studied IDI in two woody plant species, Norway spruce (*Picea abies*) and gray poplar (*Populus* × *canescens*), whose major terpenoid specialized metabolites are of different sizes. While the catalytic parameters of IDI from each species measured in vitro were in line with the different C_5_ precursor demands, the DMADP:IDP ratios of both species in vivo did not differ. Moreover, although IDI silencing in both spruce and poplar increased IDP content and significantly decreased the DMADP:IDP ratio, it caused a few significant alterations in the content of downstream terpenoid pathway intermediates or products. Taken together, these results suggest that IDI exercises a limited control over the relative amounts of different size terpenoid products. Nevertheless, the elevated IDP content of both transgenic spruce and poplar lines was associated with dramatically increased emission of isoprenol and isoprenyl acetate. Feeding experiments with cultured poplar plants indicated that these metabolites were derived directly from IDP, and their formation could serve as a metabolic mechanism to reduce high intracellular accumulation of IDP. Such a mechanism can be considered analogous to the formation of isoprene as a way to reduce high concentrations of DMADP.

## Teaser

Elevated concentrations of isopentenyl diphosphate in woody plants cannot be avoided by isopentenyl diphosphate isomerase, but rather through its metabolism into isoprenol and isoprenyl acetate.

## Graphical Abstract

**Graphical Abstract:**
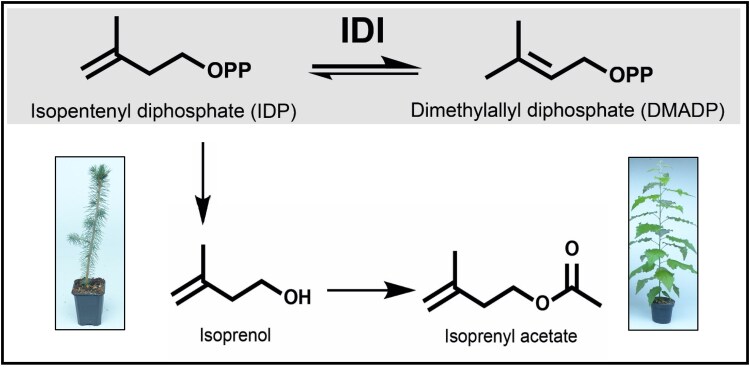

## Introduction

Terpenoids represent one of the largest and most diverse classes of natural products in plants, encompassing compounds that participate in both central metabolic processes and specialized functions. Despite their immense structural diversity, all terpenoids are derived from two universal C_5_ building blocks: dimethylallyl diphosphate (DMADP), which acts as a starter unit, and its isomer, isopentenyl diphosphate (IDP), which serves as an extender unit (Bohlmann and Keeling 2008; Hoshino and Gaucher 2018). Through sequential condensation of DMADP with one, two, or three molecules of IDP, plants produce the longer-chain prenyl diphosphates—geranyl diphosphate (GDP, C_10_), farnesyl diphosphate (FDP, C_15_), and geranylgeranyl diphosphate (GGDP, C_20_). These intermediates are central precursors for a wide array of terpenoid end products, including plant hormones (eg, gibberellins, abscisic acid, and strigolactones), sterols, photosynthetic pigments (chlorophylls and carotenoids), and volatile mono- and sesqui-terpenes. As such, terpenoids are fundamental to plant growth, development, and stress adaptation, as well as in mediating ecological interactions such as defense against herbivores and pathogens (Gershenzon and Dudareva 2007).

In plants, the synthesis of DMADP and IDP occurs via two compartmentalized pathways. The plastid-localized methylerythritol phosphate (MEP) pathway produces both C_5_ isomers via the activity of (E)-4-hydroxy-3-methylbut-2-en-1-yl diphosphate reductase (HDR, EC 1.17.7.4) in the final step. In contrast, the cytosolic mevalonate (MEV) pathway produces only IDP (McGarvey and Croteau 1995; Rohmer et al. 1996; Pulido et al. 2012; Krause et al. 2023; Sahu et al. 2023; Pérez-Gil et al. 2024). Both pathways converge through the activity of isopentenyl diphosphate isomerase (IDI; EC 5.3.3.2), which catalyzes the reversible interconversion between DMADP and IDP. Several IDI isoforms have been identified in plants, and evidence suggests that they are localized in plastids, mitochondria, peroxisomes, and potentially the cytosol (Nakamura et al. 2001; Phillips et al. 2008; Sapir-Mir et al. 2008; Okada et al. 2008; Jin et al. 2020). The number of IDI-encoding genes varies among plant species. Arabidopsis, tomato, tobacco, and rice each have two IDI genes, whereas *Catharanthus roseus* has only one (Nakamura et al. 2001; Phillips et al. 2008; Guirimand et al. 2012; Pankratov et al. 2016; Jin et al. 2020). These genes produce isoforms of differing lengths and subcellular localization; however, the precise number and compartmentation of these isoforms is unknown. Since its initial biochemical description by Lynen and colleagues in 1959 (Lynen et al. 1959), IDI has emerged as a pivotal target in metabolic engineering efforts to boost terpenoid production in heterologous systems (Berthelot et al. 2012). However, a few studies have examined heterologously expressed plant IDIs, primarily focusing on the conversion of IDP to DMADP (Agranoff et al. 1960; Ramos-Valdivia et al. 1997; Berthelot et al. 2016). The development of quantitative analytical methods for direct substrate–product detection (Krause et al. 2020) provides opportunities for more detailed kinetic and mechanistic characterization of plant IDIs. Functional studies have further demonstrated that IDI expression significantly influences plant terpenoid metabolism. For example, overexpressing IDI transcripts has been associated with altered terpenoid profiles and increased accumulation of specialized metabolites (Chen et al. 2012; Ma et al. 2017). Conversely, silencing or knocking out IDI genes has been shown to disrupt chlorophyll biosynthesis, induce pale-green phenotypes in *Nicotiana benthamiana* (Page et al. 2004), and cause lethality when completely abolished (Phillips et al. 2008). Taken together, these findings underscore the pivotal regulatory function of IDI in maintaining the equilibrium of the DMADP-IDP pool and consequently impacting both primary and secondary terpenoid biosynthesis. However, an integrated study combining the biochemical characterization of IDIs and in vivo functional analysis is lacking.

**Figure 1:**
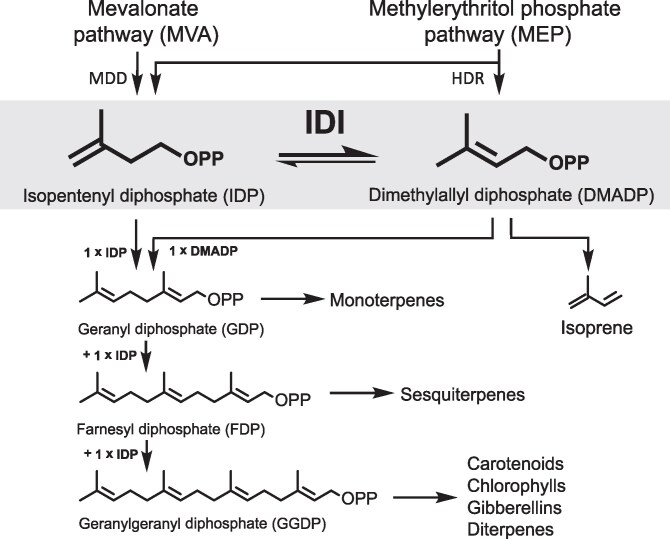
Metabolic role of isopentenyl diphosphate isomerase (IDI) and its gene expression in spruce and poplar. The basic intermediates of terpenoid chain elongation, isopentenyl diphosphate (IDP), and dimethylallyl diphosphate (DMADP) are derived from the mevalonate (MVA) and methylerythritol phosphate (MEP) pathways and are equilibrated by IDI enzyme activity. Sequential condensation of DMADP with up to three IDP molecules leads to the formation of geranyl- (GDP), farnesyl- (FDP), and geranylgeranyl diphosphate (GGDP). Larger intermediates require greater proportions of IDP to DMADP. These prenyl diphosphates can be further metabolized to a enormous variety of terpene products. DMADP can also be metabolized by dephosphorylation to isoprene, but no analogous reaction has been described for IDP. The last step of the methylerythritol phosphate pathway, HDR ((*E*)-4-hydroxy-3-methylbut-2-en-1-yl diphosphate reductase), produces a mixture of both IDP and DMADP. The final step of the mevalonate pathway, MDD (mevalonate-5-diphosphate decarboxylase), produces only IDP.

One metabolite directly linked to IDI activity is isoprene, a hemiterpene synthesized from DMADP by the enzyme isoprene synthase (IS; EC 4.2.3.27). Isoprene is the most abundant biogenic volatile organic compound emitted by terrestrial vegetation and has been the subject of intensive research for over six decades (Sharkey and Monson 2017). Despite its prevalence, the precise physiological role of isoprene in plants is unclear, and it must be explained why only perennial terrestrial plants and only about 20% of these emit this compound, particularly woody species (Monson et al. 2013; Loreto and Fineschi 2015). Several hypotheses have been proposed regarding the functional significance of isoprene emission. Empirical evidence supports its role in providing thermal protection (Sharkey and Singsaas 1995; Behnke et al. 2007; Velikova et al. 2011; Pollastri et al. 2019) and mitigating oxidative stress induced by high-light conditions during photosynthesis (Affek and Yakir 2002; Penuelas et al. 2005; Velikova et al. 2005; Vickers et al. 2009). Additionally, recent studies have suggested that isoprene may have signaling functions, including hormone-like activity (Zuo et al. 2019; Miloradovic van Doorn et al. 2020), promotion of flowering (Terry et al. 1995), and functioning as a semiochemical involved in plant-insect communication (Loivamaeki et al. 2008). From a metabolic perspective, isoprene formation may serve as a mechanism to recycle inorganic phosphate from the DMADP pool that accumulates during periods of high photosynthetic and carbon assimilatory activity (Rosenstiel et al. 2004). This hypothesis is supported by observations that isoprene emission predominantly occurs in actively photosynthetic leaves (Rasulov et al. 2014) and is especially pronounced in fast-growing tree species (Dani et al. 2014). Kinetic analyses have shown that isoprene synthase has a much lower affinity for DMADP than prenyltransferases that catalyze the formation of GDP, FDP, and GGDP. This ensures that DMADP is only directed toward isoprene production when intracellular concentrations are high (Fischbach et al. 2000; Wolfertz et al. 2004; Schnitzler et al. 2005). Heterologous expression of isoprene synthase in the non-emitting species *Arabidopsis thaliana* resulted in enhanced growth (Loivamaeki et al. 2007). In contrast, suppression of isoprene emission in transgenic poplar (Populus spp.) caused only minor alterations in central metabolism (Behnke et al. 2007, 2010). These findings suggest that, while not essential for primary metabolism, isoprene emission may contribute to physiological resilience under environmental stress. The enzymatic conversion of DMADP to the non-phosphorylated isoprene raises the possibility of analogous reactions involving IDP. IDP has indeed been shown to be converted into 3-methyl-3-buten-1-ol (isoprenol) via nonspecific phosphohydrolase activity in *Escherichia coli* (Chou and Keasling 2012). Isoprenol and its acetylated derivative, 3-methyl-3-buten-1-yl acetate (isoprenyl acetate), have been identified as minor volatile components of certain ripe fruits (Sampaio and Nogueira 2006; Steingass et al. 2015). However, the metabolic origins, enzymatic pathways, and physiological significance of these compounds remain poorly understood (Fig. 1).

In this study, we aimed to elucidate both the biochemical properties and in vivo functional roles of IDI in woody plants because these often produce larger quantities of more diverse specialized isoprenoid products, such as resin, rubbers, and volatiles, compared to Arabidopsis and most other herbaceous plants. The greater output and size variation of terpenoid metabolites in woody plants may require more precise control of the ratio between the starter (DMADP) and extender (IDP) units of terpenoid condensation, whose interconversion is catalyzed by IDI. We chose to study *Picea abies* (Norway spruce) and *Populus* × *canescens* (gray poplar, a hybrid of *P. tremula* and *P. alba*) because of their contrasting demands for terpenoid biosynthetic units. The conifer *P. abies* produces high amounts of mono- and diterpenoid oleoresins (C_10_ and C_20_ compounds), which require relatively large quantities of extender (IDP) to starter (DMADP) units, but emits only trace levels of isoprene (C_5_) (Martin et al. 2002; Keeling and Bohlmann 2006). In contrast, gray poplar emits large quantities of isoprene, which requires only DMADP as a precursor, and produces very little terpenoid oleoresin. These contrasting metabolic phenotypes imply differing cellular demands for DMADP and IDP. By regulating the ratio of these two C_5_ building blocks, plants may be able to favor the formation of different sizes of terpenoid metabolites (Bergman et al. 2024). We hypothesized that IDI from *P. abies* and *P. × canescens* would exhibit distinct kinetic and regulatory properties reflecting these metabolic differences. To test this hypothesis, we conducted in vitro biochemical characterization of IDI enzymes from both species, followed by *in planta* functional analysis through transgenic manipulation of IDI expression. Plants were transformed to either silence or overexpress IDI, and the resulting changes in terpenoid metabolism were analyzed. Notably, we observed a marked increase in the emission of isoprenol and isoprenyl acetate under conditions of elevated IDP concentration, revealing a potential previously unknown mechanism for maintaining IDP homeostasis in plant cells.

## Results

### Spruce and poplar each contain a single *IDI* gene copy

One copy of *IDI* was found in both *P.* × *canescens* and *P. abies* (termed *PcIDI* and *PaIDI*, respectively). The genes of each species appeared to contain a transit peptide for plastidial localization, based on predictions using several bioinformatic algorithms, while the sequence starting with the second methionine of the open reading frame was predicted to be localized in the plastids, peroxisome, or cytoplasm, depending on the algorithm (Figure S1a–1c). The amino acid level identity between *PcIDI* and *PaIDI* with and without transit peptide is 67% and 81%, respectively. Characteristic sequence motifs of the IDI enzyme family were present, including NxxCxHP (nucleotide 171) and ExE (nucleotide 234) (Berthelot et al. 2012). Because Arabidopsis, tomato, tobacco, and rice have been thought to possess a second functional IDI (Nakamura et al. 2001; Phillips et al. 2008; Pankratov et al. 2016; Jin et al. 2020), *P*. × *canescens* was intensively screened for a homolog using a transcriptomic approach, but no additional sequences were found.

*PcIDI* gene expression in poplar was highest in leaves, followed by root and stem tissue. Upon herbivory, *PcIDI* gene expression was significantly increased in leaf tissue but was not significantly affected by jasmonic acid (JA) treatment. In spruce, *PaIDI* was mostly expressed in needle and root tissue, and less in stem tissue. Upon methyl jasmonate treatment, all tissues showed a significant increase in *IDI* expression (Figure S2).

### The kinetic properties of the spruce and poplar IDIs are distinct

**Figure 2:**
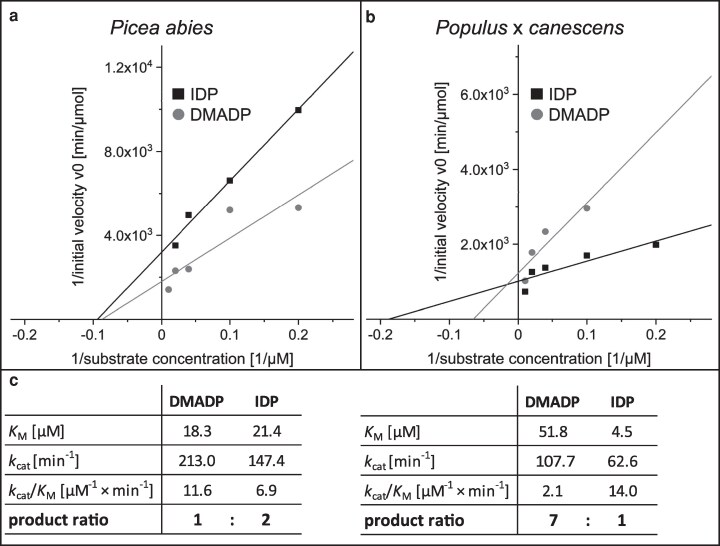
Kinetic analysis of heterologously expressed recombinant IDI enzymes of poplar and spruce. Lineweaver-Burk plots of spruce *Pa*IDI and poplar *Pc*IDI for the substrates DMADP and IDP. a, b) Summary of the kinetic parameters. c) Each data point represents the mean of two technical replicates from two separate experiments.

To measure the kinetic properties of spruce and poplar IDI for the 2 substrates, DMADP and IDP, heterologously expressed proteins were purified and assayed under optimized pH and temperature conditions (Fig. 2a and 2b and Figures S3a and 3b and S4). *Pa*IDI (from spruce) showed similar affinities for both substrates with *K*_M_ values of 21.4 µM for IDP and 18.3 µM for DMADP. Catalytic turnover favored the production of IDP, yielding a DMADP:IDP ratio under equilibrium conditions of 1:2. In contrast, *Pc*IDI (from poplar) showed a higher affinity for IDP than for DMADP with *K*_M_ values of 4.5 µM and 51.8 µM, respectively. This resulted in a higher catalytic turnover (*k*_cat_/*K*_M_) for IDP compared to DMADP, and the DMADP:IDP ratio under equilibrium conditions was 7:1 (Fig. 2c).

### Silencing *PaIDI* in spruce increases IDP levels over 10-fold and decreases monoterpene and sesquiterpene content

**Figure 3:**
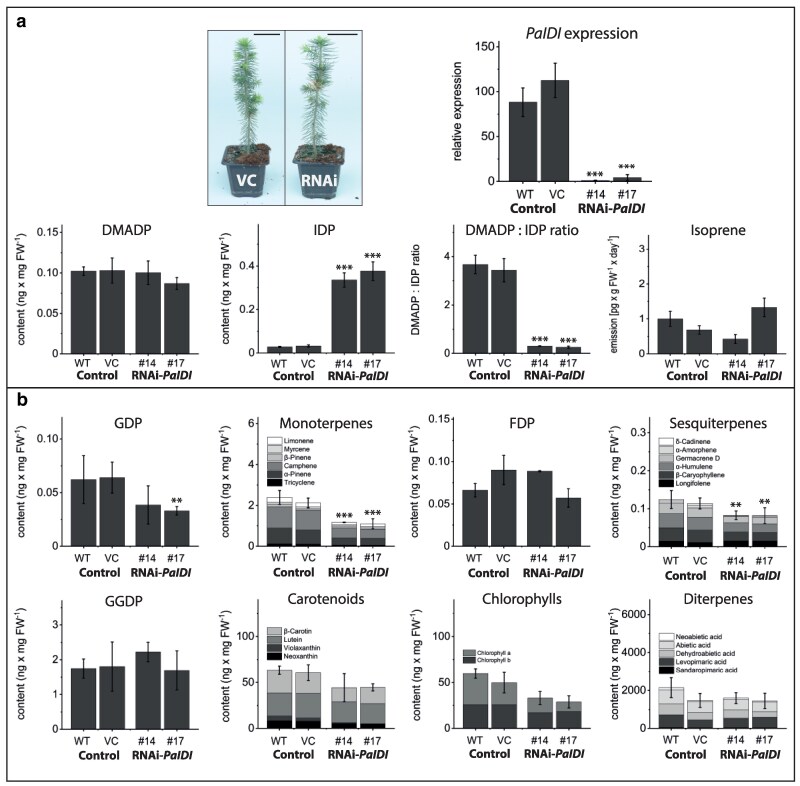
Analysis of terpenoid metabolites in needles of Norway spruce (*Picea abies*) with silenced isopentenyl diphosphate isomerase (*PaIDI)* expression. Transgenic spruce with silenced (RNAi) *PaIDI* expression showed similar morphology as the vector control (VC), transformed with an empty vector. Scale bars represent 5 cm. Isopentenyl diphosphate (IDP) content increased in silenced lines, but not dimethylallyl diphosphate (DMADP) content, which decreased the DMADP:IDP ratio. The emission of isoprene from saplings was also not affected by silencing. a) Geranyl diphosphate (GDP) content was significantly decreased in one transgenic line with a tendency towards reduction in the other. Reductions were also observed in monoterpenes and sesquiterpenes, but not in other metabolites measured. b) Prenyl diphosphates were analyzed by LC-MS/MS, and isoprene by GC-MS of trapped headspace volatiles. Monoterpenes, sesquiterpenes, and diterpenes extracted from internal pools were analyzed by GC-MS, and carotenoids and chlorophylls by HPLC with UV detection. Values are given as mean ± standard deviation of at least three biological replicates per line. *** = *P* < 0.001; * = *P* < 0.05; Student's *t*-test of individual lines vs. vector controls. For *P*-values, see Table S1. Abbreviations: IDP, isopentenyl diphosphate; DMADP, dimethylallyl diphosphate; GDP, geranyl diphosphate; FDP, farnesyl diphosphate; GGDP, geranylgeranyl diphosphate.

In order to test if IDI is involved in the regulation of terpenoid biosynthesis by its control of DMADP and IDP availability, transgenic Norway spruce plants with suppressed levels of *IDI* gene expression were generated. IDI transcript levels were reduced by over 95%, but no obvious morphological differences occurred in transgenic lines. While DMADP concentration was not affected by *IDI* silencing, IDP concentration was increased by over ten-fold compared to the unsilenced controls. This also affected the DMADP:IDP ratio, which was reduced from 4:1 to 0.2:1 (Fig. 3a, Table S1). The content of geranyl diphosphate (GDP) and its corresponding monoterpenoid products were significantly reduced by about 50% upon *IDI* silencing. Sesquiterpenoids were also significantly reduced in a similar fashion. The levels of geranylgeranyl diphosphate (GGDP), carotenoids, chlorophylls, diterpene resin acids, and isoprene were not altered in transgenic spruce plants (Fig. 3b, Table S1).

### Silencing and overexpressing *IDI* in poplar altered DMADP:IDP ratios but had almost no effect on downstream biosynthetic intermediates and products

**Figure 4:**
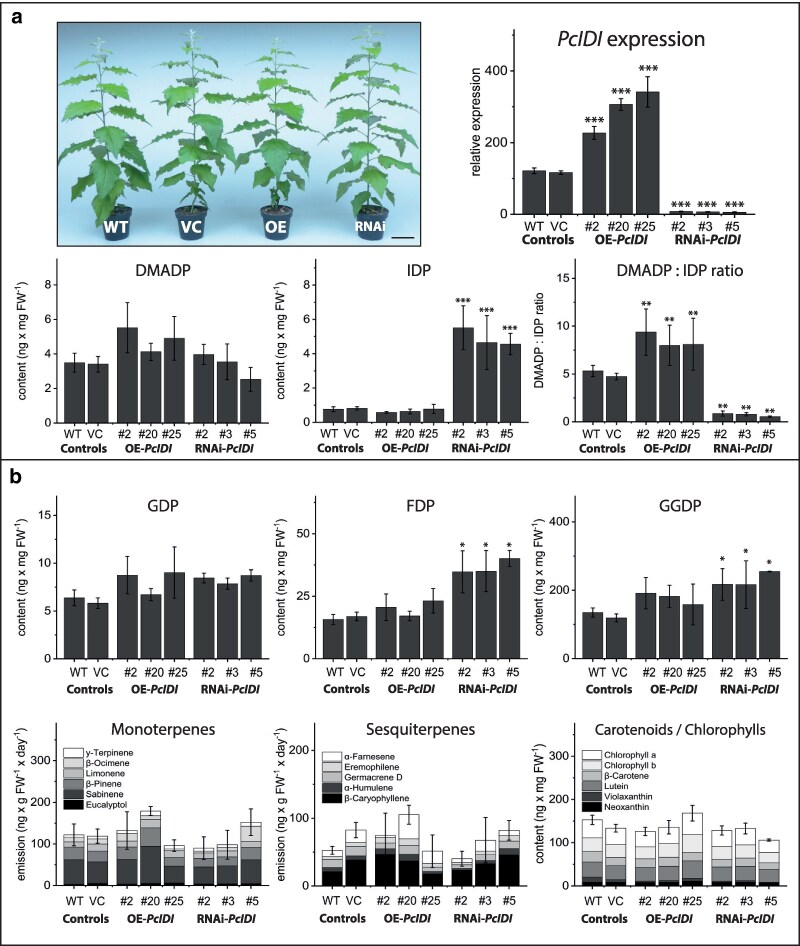
Analysis of terpenoid metabolites of gray poplar (*Populus* × *canescens*) lines with overexpressed and silenced isopentenyl diphosphate isomerase (*P*c*IDI*). Scale bar represent 10 cm.Transgenic poplar with overexpressed (OE) or silenced (RNAi) *PcIDI* showed similar morphology as wild-type control lines (WT) or vector controls (VC), transformed with an empty vector. Overexpression significantly increased the dimethylallyl diphosphate:isopentenyl diphosphate (DMADP:IDP) ratio in leaves, while silencing increased the IDP content and decreased the DMADP:IDP ratio. a) *PcIDI* silencing resulted in an increase in farnesyl diphosphate (FDP) content in leaves, but geranyl diphosphate (GDP) and geranylgeranyl diphosphate (GGDP) were not affected, and none of these prenyl diphosphates were affected by overexpression of *PcIDI*. Emission of mono- and sesquiterpenes from saplings as well as the content of chlorophylls and carotenoids in leaves was also not affected by transformation. b) All compounds were analyzed as described in Fig. 3. Values are given as mean ± standard deviation of at least four biological replicates per line, measured in technical duplicates. *** = *P* < 0.001; ** = *P* < 0.01; * = *P* < 0.05; Student's *t*-test of individual lines vs. vector controls. For *P*-values see Table S2. Abbreviations: IDP, isopentenyl diphosphate; DMADP, dimethylallyl diphosphate; GDP, geranyl diphosphate; FDP, farnesyl diphosphate; GGDP, geranylgeranyl diphosphate.

As for spruce, transgenic poplar plants silenced in *IDI* (over 95% by RNAi, or overexpressing (OE) *IDI* by 2-fold to 3-fold) showed no morphological differences from control plants. In addition, DMADP concentration was not significantly altered. IDP concentrations, however, were increased about ten-fold in RNAi-*IDI* lines with a resulting decrease in DMADP:IDP ratio. IDP concentrations were not significantly changed in OE-*IDI* lines, but the DMADP:IDP ratio nearly doubled (Fig. 4a, Table S2). Altering the proportions of these C_5_ intermediates had only small effects on the content of other prenyl diphosphates in poplar, with only FDP content elevated in RNAi-*IDI* lines by about two-fold. However, higher levels of FDP did not lead to increased emission of sesquiterpenoids or an increase in the accumulation of the main sterol ß-sitosterol (Figure S5). Additionally, emission of monoterpenoids as well as the content of carotenoids and chlorophylls were not affected by altered *IDI* gene expression (Fig. 4b, Table S2). To test if the metabolic alterations in RNAi-*PcIDI* lines would have a feedback effect on the supply of prenyl diphosphates, we measured the flux through the MEP pathway using a ^13^C-label derived from the incorporation of ^13^CO_2_ via photosynthesis through the pathway and into isoprene. However, flux was not significantly different from that of control unsilenced plants (Figures S6 and S7).

### *IDI*-silenced spruce and poplar both emit unexpected C_5_ volatiles

**Figure 5:**
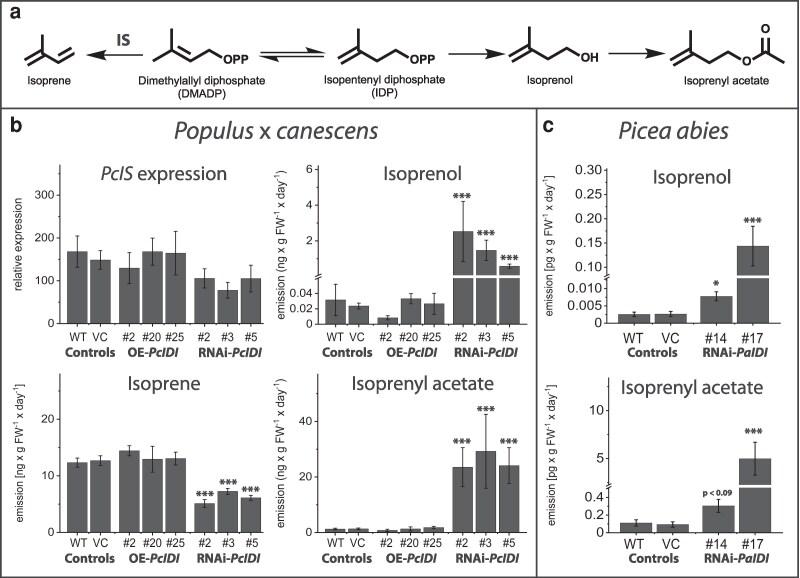
Isoprenol and isoprenyl acetate emission from transgenic lines of poplar and spruce saplings. DMADP is converted to isoprene in a reaction catalyzed by isoprene synthase (IS). Loss of OPP generates a carbocation that is rearranged and deprotonated to form isoprene. IDP likely gives rise to isoprenol and isoprenyl acetate by successive dephosphorylation and acetylation. a) Headspace volatiles of poplar collected on Carbotrap-X adsorbent and analyzed by GC-MS showed significant increases in isoprenol and isoprenyl acetate emission in *IDI*-silenced lines. Overexpression of the *PcIDI* gene did not affect these volatiles in poplar. b) Isoprenol and isoprenyl acetate were significantly increased in *PaIDI*-silenced spruce lines compared to the controls. c) Values are given as mean ± standard deviation of at least four biological replicates per line. * = *P* < 0.05, *** = *P* < 0.001; Statistical analysis were performed by using Student's *t*-test of individual lines vs. vector controls. For *P*-values, see Table S3. Abbreviations: IDP, isopentenyl diphosphate; DMADP, dimethylallyl diphosphate; IS, isoprene synthase.

The emission of isoprene, a major volatile of poplar, was reduced by about half in RNAi-*IDI* lines, while there was no change in OE-*IDI* lines. The significant changes in isoprene emission upon *IDI* manipulation prompted us to look for changes in other volatiles. However, GC-MS analysis detected the presence of two unexpected C_5_ volatiles, identified as 3-methyl-3-buten-1-ol (isoprenol) and 3-methyl-3-buten-1-yl acetate (isoprenyl acetate) by comparison of their retention times and mass spectra with those of authentic standards. Both volatiles were found to be emitted from transgenic RNAi-*IDI* poplar plants at rates several orders of magnitude higher than those of controls (Fig. 5a and 5b, Table S3). Screening of Norway spruce RNAi-*IDI* plants showed that isoprenol and isoprenyl acetate were also emitted at much higher rates than from the controls (Figure 5c, Table S3).

### Silencing of *IDI* together with *HDR* overexpression in poplar further increased IDP levels, which increased prenyl diphosphate intermediate, carotenoid, and chlorophyll levels, and isoprenol and isoprenyl acetate emission

**Figure 6:**
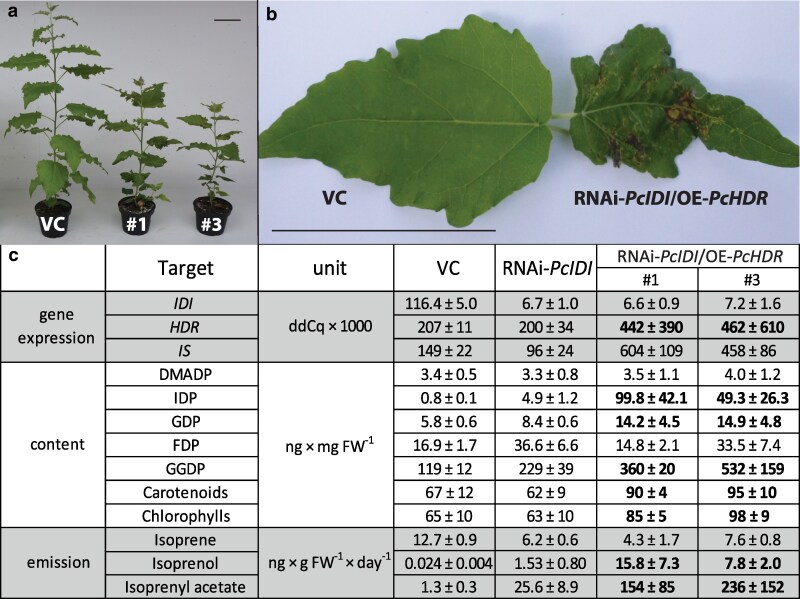
Analysis of gene expression and terpene metabolites in poplar lines with silenced *PcIDI* in combination with overexpression of *PcHDR*. Scale bars represent 10 cm. Double transgenic lines (#1 and #3) showed reduced growth and development compared to vector control (VC) plants transformed with an empty vector. a) Leaves of double transgenic plants showed necrotic spots and impaired development. b) Gene expression, terpene content, and emissions in double transgenic lines (RNAi-*PcIDI*/OE-*PcHDR*) are compared to vector controls (VC) and single *PcIDI*-knock-down lines (RNAi-*PcIDI*). IDP increased up to approximately 100-fold in double transgenic lines, while DMADP was not affected. The larger prenyl diphosphates, GDP and GGDP, and the content of carotenoids and chlorophylls increased 1.5-fold to 4.5-fold. Emissions of isoprenol and isoprenyl acetate increased dramatically up to 500-fold in double transgenic lines, while isoprene remained unaffected. c) Values are given as mean ± standard deviation of four biological replicates per line. Statistical significance was tested using Student's *t*-test. Values for the double knock-down lines that were significantly different from those for the RNAi-*PcIDI* single knock-down are shown in bold. For *P*-values, see Table S4. Abbreviations: IDP, isopentenyl diphosphate; DMADP, dimethylallyl diphosphate; GDP, geranyl diphosphate; FDP, farnesyl diphosphate; and GGDP, geranylgeranyl diphosphate.

Given the minor changes in downstream metabolites after *IDI* silencing, we sought to disrupt the balance between DMADP and IDP even further to assess the effects on terpenoid metabolism. Thus, we transformed poplar by overexpression of the last step of the MEP pathway, (*E*)-4-hydroxy-3-methylbut-2-en-1-yl diphosphate reductase (HDR) to increase IDP production while, at the same time, silencing *IDI*. This resulted in morphologically altered plants with impaired leaf development and necrotic spots. This phenotype appeared mostly in older leaves (LPI >#5), while younger leaves looked like those of wild type plants (Fig. 6a and 6b). IDP concentrations increased by nearly two orders of magnitude in the RNAi-*IDI*/OE-*HDR* transgenic lines compared to control plants, while DMADP content remained unaffected. The downstream prenyl diphosphate intermediates, GDP and GGDP, as well as carotenoids and chlorophylls, were significantly increased 1.5-fold to 4.5-fold in these double-transformed lines, while isoprenol and isoprenyl acetate emission were elevated by over two orders of magnitude. *IDI* gene expression was not altered compared to RNAi-*IDI* plants, which confirmed that the altered phenotype was caused by the additional overexpression of the *HDR* gene (Fig. 6c, Table S4).

### The isoprenol and isoprenyl acetate emitted from poplar are formed from IDP

**Figure 7:**
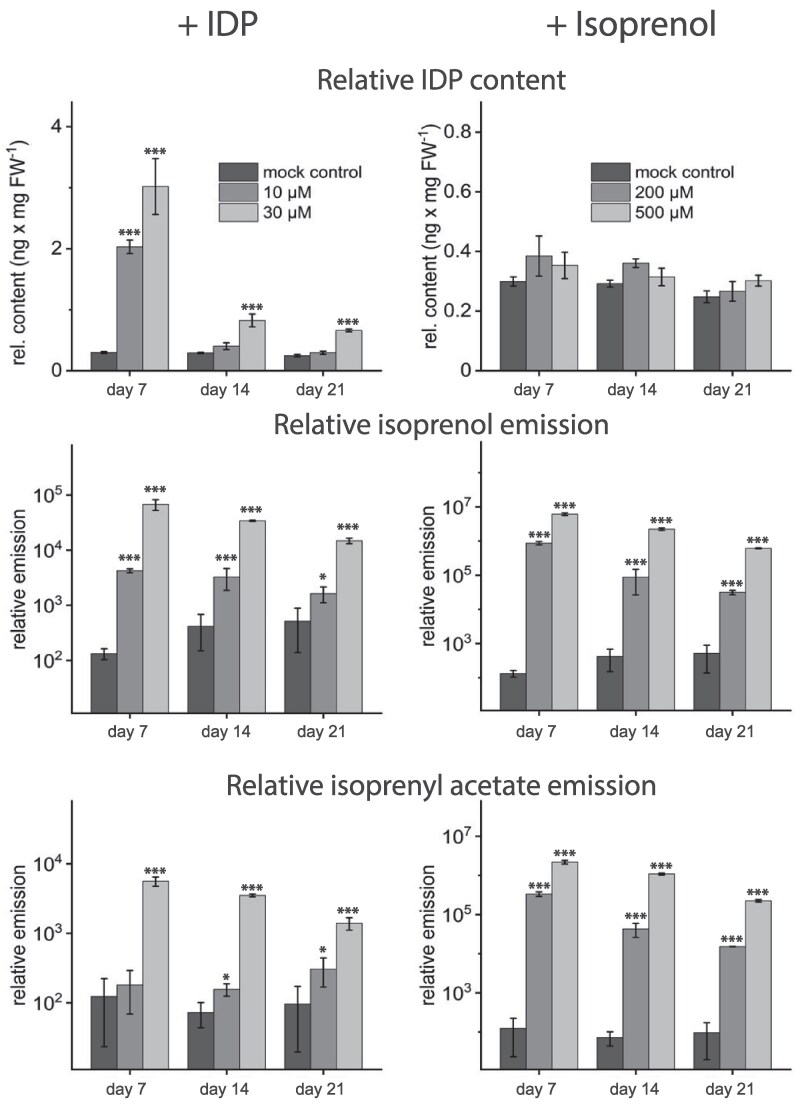
IDP content and emission of isoprenol and isoprenyl acetate from poplar saplings supplemented with IDP or isoprenol. Young poplar saplings were planted in medium supplemented with 10 µM or 30 µM IDP, or 200 µM or 500 µM isoprenol and volatiles were measured for 24 h after 7, 14, and 21 days using polydimethylsiloxane (PDMS) tubing, followed by GC/MS analysis. Supplementation with IDP caused a significant increase of internal IDP content relative to the amount supplied in the medium. IDP supplementation also caused a significantly increased emission of isoprenol and isoprenyl acetate. Supplementation of isoprenol did not affect IDP concentration but caused significantly increased emission of isoprenyl acetate. The emission of isoprenol and isoprenyl acetate and the content of IDP were all expressed in relative terms and normalized by tissue weight to compare the results of volatile collection and tissue extraction. Emission of isoprenol and isoprenyl acetate are depicted on a log_10_ scaled axis. Values are given as mean ± standard deviation of at least four biological replicates per line and are measured in relation to the controls. * = *P* < 0.05; *** = *P* < 0.001; Statistical analysis were performed by using Student's *t*-test. For *P*-values, see Table S5.

The structural similarity between isoprenol and isoprenyl acetate to IDP, as well as the accumulation of all these compounds in transgenic poplar, and spruce with silenced *IDI* gene expression levels suggested their direct formation from IDP. To gain more information about the pathway of isoprenol and isoprenyl acetate formation, IDP was administered to *P*. × *canescens* wild-type plants by addition to solid growth medium (Fig. 7, Table S5). Over 21 days, the IDP content of control lines relative to DMADP was stable at 0.29 ng × mg FW^−1^. For example, supplementation of 30 µM IDP yielded significantly increased internal IDP throughout the course of the experiment, showing the highest content at day 7 (3.02 ng × mg FW^−1^), which decreased to 0.80 and 0.66 ng × mg FW^−1^ at days 14 and 21, respectively. This indicates an uptake of the supplemented IDP by the plant and incorporation into internal pools.

The IDP taken up appeared to be converted directly to isoprenol and isoprenyl acetate based on the patterns of emission of these two C_5_ volatiles. For example, on day 7, emission of isoprenol was significantly increased by a factor of 500 with 30 µM IDP, compared to plants without IDP. The emission of isoprenyl acetate followed a similar pattern when 30 µM of IDP were supplemented in the medium.

To check whether either a back reaction from isoprenol to IDP is possible or isoprenol is directly converted to isoprenyl acetate, another feeding experiment was performed with the medium supplemented with 200 and 500 µM isoprenol. IDP content was similar in plants fed with isoprenol compared to mock controls, indicating that no phosphorylation of isoprenol to IDP occurred. The emission of isoprenyl acetate after feeding isoprenol exceeded that of the controls by a factor of 2,700, 600, and 150 on days 7, 14, and 21 after feeding 200 µM to the medium. Plant volatile profiles were also screened for the DMADP analogs of isoprenol and isoprenyl acetate, 3-methyl-2-buten-1-ol (prenol), and 3-methyl-2-buten-1-yl acetate (prenyl acetate), but these were not detected.

To check if the supplementation of IDP and isoprenol and the corresponding production of volatiles was influenced by altered gene expression at the level of DMADP and IDP, *PcHDR*1, *PcHDR*2, and *PcIDI* gene expression were analyzed using RT-qPCR, but no significant change between mock control and the different treatments was observed for any of these genes (Figure S8). Although experiments with labeled precursor compounds still need to be performed, our results support the hypothesis that isoprenol and isoprenyl acetate emitted by poplars originate from IDPs.

## Discussion

Isopentenyl diphosphate isomerase (IDI; EC 5.3.3.2) plays a central role in terpenoid biosynthesis by catalyzing the reversible interconversion between dimethylallyl diphosphate (DMADP) and isopentenyl diphosphate (IDP), the two fundamental C_5_ building blocks of all isoprenoids. Despite the simplicity of this reaction, IDI exerts a potentially critical influence on the balance between precursor supply and metabolic demand in plant terpenoid biosynthetic networks. In the present study, we investigated the biochemical characteristics and in vivo function of IDI in two woody species with contrasting terpenoid profiles: Norway spruce (*P. abies*) and gray poplar (*P. × canescens*). *P. abies* produces large quantities of terpenoid oleoresin dominated by monoterpenes (C_10_) and diterpenes (C_20_), whereas *P. × canescens* emits high levels of isoprene (C_5_). These differences reflect divergent demands for DMADP and IDP: monoterpene synthesis requires one mole of each, diterpene synthesis requires one mole of DMADP and three moles of IDP, while isoprene formation depends solely on DMADP. This contrast provided an ideal system to examine whether species-specific differences in IDI function contribute to the distinct terpenoid composition of these plants (Fig. 1).

Genomic and transcriptomic analyses revealed that both *P. abies* and *P. × canescens* possess a single IDI gene expressed in leaves, stems, and roots. This differs from species such as *Arabidopsis thaliana* and *Solanum lycopersicum*, which harbor two distinct IDI genes (Phillips et al. 2008; Sun et al. 2010). Bioinformatic predictions indicated that both spruce and poplar IDI proteins possess putative N-terminal transit peptides, suggesting plastidial localization; however, localization algorithms produced inconsistent results. Notably, both gene sequences contain multiple methionine residues near the 5′ terminus, each situated within potential Kozak consensus sequences (Kozak 1986; Hinnebusch 2011). This suggests the potential for alternative translation initiation sites, possibly leading to isoforms with different subcellular destinations. Further experimental confirmation, such as proteomic localization analyses or the use of fluorescently tagged IDI constructs, will be required to determine the precise compartmentation of these enzymes.

Biochemical characterization of recombinant spruce IDI revealed an in vitro DMADP:IDP equilibrium ratio of approximately 1:2 (Fig. 2), whereas in vivo measurements in spruce needles yielded a substantially higher ratio of 4:1. These results indicate that steady-state levels of these metabolites in planta may be determined more by downstream metabolic consumption than by enzymatic equilibrium. Or, the in vivo environment of IDI is much different than the in vitro conditions we employed for enzyme characterization. RNA interference-mediated silencing of IDI in spruce resulted in marked accumulation of IDP but did not significantly alter DMADP concentrations. Metabolite profiling of these lines revealed reduced monoterpene and sesquiterpene levels, while isoprene emission remained unchanged. Given that *P. abies* emits isoprene at only trace levels (Figs. 3 and 5) and lacks a clearly identifiable isoprene synthase (IS) gene, isoprene formation in spruce may occur through non-enzymatic processes (Brueggemann and Schnitzler 2002) or as a by-product of 2-methyl-3-buten-2-ol (MBO) synthase activity (Gray et al. 2011). The reduction in monoterpene and sesquiterpene production observed in silenced lines likely reflects an imbalance in the DMADP:IDP ratio, which decreases the relative availability of the starter unit (DMADP). The absence of significant changes in other terpene classes may be attributed to the cellular compartmentation of terpenoid metabolism; for example, monoterpene and sesquiterpene biosynthesis occurs predominantly in the epithelial cells of resin ducts (Celedon et al. 2017), where local precursor pools may differ from those in photosynthetic tissues.

In *P. × canescens*, isoprene emission represents a dominant carbon flux that relies exclusively on DMADP. Enzymatic assays demonstrated that poplar IDI maintains a strong catalytic bias toward DMADP formation, producing a DMADP:IDP ratio of 7:1 in vitro (Fig. 2). This bias corresponds closely with in vivo measurements, which revealed a ratio of approximately 5:1 in leaf tissue. Transgenic poplar plants with reduced IDI expression exhibited a significant decline in isoprene emission, whereas mono- and sesqui-terpene emission remained largely unchanged (Figs. 4 and 5). These findings indicate that IDI activity in poplar directly regulates the supply of DMADP available for isoprene synthesis but has limited influence on the biosynthesis of larger terpenoid products.

A striking and previously unreported outcome of IDI silencing in both spruce and poplar was the enhanced emission of isoprenol (3-methyl-3-buten-1-ol) and isoprenyl acetate (3-methyl-3-buten-1-yl acetate). These emissions correlated with elevated IDP levels, suggesting that these volatiles derive directly from excess IDP. Feeding experiments confirmed this relationship: exogenous IDP supplementation increased both isoprenol and isoprenyl acetate emissions, and isoprenol feeding specifically enhanced isoprenyl acetate formation (Fig. 7). The unaltered levels of other terpenoid metabolites during these treatments imply a direct and immediate conversion pathway from IDP to these volatiles.

To further increase IDP accumulation, poplar plants were co-transformed with constructs that simultaneously silenced IDI and overexpressed HDR, the enzyme catalyzing the final step of the MEP pathway. These double-transgenic lines displayed stunted growth, leaf deformities, and elevated levels of prenyl diphosphates and photosynthetic pigments, along with dramatically increased emissions of isoprenol and isoprenyl acetate (Fig. 6). Although isoprenol and isoprenyl acetate have been previously identified in the volatile blends of certain ripe fruits, including pineapple and mangaba (Sampaio and Nogueira 2006; Steingass et al. 2015), their physiological significance in plants remains unclear. Our results suggest that their production functions as a metabolic safety mechanism, mitigating potentially toxic accumulation of IDP (Georg et al. 2018) and facilitating phosphate recovery for other biosynthetic processes. These volatiles may also contribute to protection against thermal and oxidative stress, as previously proposed for isoprene and monoterpenes (Sharkey 2005; Vickers et al. 2009; Shan and Jin 2025).

Additional mechanisms for regulating IDP homeostasis may exist in plants. For example, under nitrate starvation, Arabidopsis roots accumulate glycosylated derivatives of MEP pathway intermediates (Ward et al. 2011), and similar conjugates have been identified for other isoprenoid precursors (Gonzales-Cabanelas et al. 2015). While such glycosides have not yet been reported in spruce or poplar, they may represent an alternative strategy to buffer fluctuations in terpenoid precursor pools under environmental or developmental stress.

In conclusion, our findings demonstrate that IDI from spruce and poplar exerts only limited regulatory control over terpenoid biosynthesis. Despite the distinct in vitro kinetic properties and product ratios of the two enzymes, both species have comparable DMADP:IDP ratios in vivo, suggesting that metabolic flux control is primarily exerted downstream of IDI. Silencing of IDI in both species caused substantial IDP accumulation and a few alterations in downstream terpenoid metabolites, indicating that IDI does not directly determine the compositional divergence of terpenoid products between the two species (Figure S9).

Importantly, our study identifies the formation and emission of isoprenol and isoprenyl acetate as a previously unknown metabolic release mechanism that alleviates excess IDP accumulation—analogous to the conversion of DMADP to isoprene described in other species (Rosenstiel et al. 2004). Such a process for excess IDP metabolism may operate in all plants regardless of life form or whether or not they emit isoprene. We propose that these IDP-derived volatiles function not only as overflow metabolites but potentially as signaling molecules, reflecting the rate of terpenoid flux and coordinating cellular responses to metabolic or environmental stress. Increasing evidence supports a signaling role for isoprene (Zuo et al. 2019); similar functions for isoprenol and isoprenyl acetate may represent an unrecognized layer of metabolic regulation in plants.

## Materials and methods

### Plant and insect cultures

*Populus × canescens* trees (clone INRA 7171-B4) were propagated and grown in a greenhouse (24 °C, 60% relative humidity, 100 µmol × m^−2^ × s^−1^ PAR, and 16 h/8 h light/dark) in a 1:1 mixture of sand and soil (Klasmann-Deilmann, Geeste, Germany), until they reached a height of 1 m. After whole plant measurements, such as volatile collection, a single leaf (LPI #7 according to methods described in a previous study (Frost et al. 2007)) was detached and flash-frozen in liquid nitrogen for metabolite and gene transcript analyses. Four plants were used for each wild type and transgenic line, except for empty vector controls, which are represented by three lines with four plants each.

*Picea abies* saplings propagated from clone 3369-Schongau (Samenklenge and Pflanzgarten, Laufen, Germany) were used for methyl jasmonate (MJ) treatment or transgenic saplings propagated from an embryogenic culture of *P. abies* clone 186/3c VIII were used for biochemical characterization (kindly provided by Harald Kvaalen, Norwegian Institute of Bioeconomy Research). Both clones were grown with altered summer and winter conditions. Under summer conditions, saplings were grown in standard soil under a 21 °C-day/16 °C-night temperature cycle, controlled light conditions (16 h per day at 150 to 250 µmol × m^−2^ × s^−1^, obtained from a mixture of cool-white fluorescent and incandescent lamps), and a relative humidity of 70% in a climate chamber (Weiss Technik GmbH (Voetsch), Reiskirchen, Germany). In an 8-week winter period, the temperature during day and night was constant at 6 °C, with light for only 8 h per day. For characterization, two-year old saplings with a size of 15 ± 5 cm were detached 0.5 cm aboveground and flash-frozen in liquid nitrogen. Needles were separated by carefully breaking them from the frozen stem, ground to a fine powder, and stored at −80 °C.

*Chrysomela populi* leaf beetles and their egg clutches were collected in a poplar common garden established in Jena, Germany (50°56′36.6″ N; 11°32′59.0″ E) and reared under lab conditions for several generations.

### Plant treatments

Jasmonic acid (JA) treatment: A 100 µM solution of (±)-JA (stock: 500 mg × mL^−1^ in ethanol; Cayman Chemical, Ann Arbor, MI, USA) in water was used to test for altered *IDI* gene expression caused by induction of plant defenses. A solution of 0.02% ethanol served as a control. Leaves of *P. × canescens* wild type trees (1 m in height) were detached and placed in 10 mL glass vials filled with JA or control solution for 24 hours. In total, 13 leaves were used per treatment. Leaves were flash-frozen in liquid nitrogen afterwards and stored at −80 °C until further analysis.

*C. populi* herbivory: Beetles were starved for two days before the experiment, and three individuals were placed in a petri dish with two detached leaves for three days. Leaves were wrapped with wet paper and aluminum foil to reduce water loss. Leaves were flash frozen in liquid nitrogen at the end of the experiment and stored at −80 °C until further analysis. Six biological replicates were used.

Methyl jasmonate (MJ) treatment: For MJ treatment, four biological replicates of two-year-old spruce plants were sprayed with 1 mM MJ solution. Samples were collected before and after two days of treatment, ground to a fine powder, and stored at −80 °C.

### IDI sequences from poplar and spruce

Based on the published sequences from *Populus trichocarpa* and *Picea glauca*, IDI amino acid and gene sequences from *P. × canescens* and *P. abies* were obtained from NCBI and PlantGenIE databases via BLAST search analyses from transcriptome data. NCBI gene accession and PlantGenIE numbers for *P. trichocarpa* IDI were XM_002325433 and Potri.019G053700, respectively, and the coding sequence of the *PcIDI* gene was verified by subcloning and sequencing to be identical to that of *P. trichocarpa*. The NCBI gene accession number for *P. glauca IDI* was BT108967. and a partial sequence of *PaIDI* was available in the PlantGenIE database, having the number MA_60222g0010. Extraction and sequencing of the *IDI* gene from *P. abies* revealed several differences from *P. glauca* IDI.

The DNAStar Lasergene program version 13.0 (MegAlign) was used to align and to calculate the deduced amino acid sequences of each full-length cDNA or of known sequences from other gymnosperms and angiosperms. The amino acid alignment was conducted by use of ClustalW (Gonnet 250 matrix, gap penalty 10.00, gap length penalty 0.20, delay divergent sequences 30%, gap length 0.10, DNA transition weight 0.5). Screening for intracellular localization sequences used the web-based tools iPSORT (https://ipsort.hgc.jp), Predotar (https://urgi.versailles.inra.fr/predotar), TargetP (https://services.healthtech.dtu.dk/services/TargetP-2.0), PTS predictor (https://mendel.imp.ac.at/pts1), Wolf PSORT (https://www.genscript.com/wolf-psort.html), and DeepLoc21 https://services.healthtech.dtu.dk/services/DeepLoc-2.1. RNA-sequencing analysis from wild-type *P.* × *canescens* plants were performed to screen for a putative second *IDI* gene or for a gene that lacks the putative transit peptide (for the generation of the data, see below). To map the gene sequences against the transcriptome and obtain reads per kilobase million (RPKM) values, the CLC Genomic Workbench 12.0.3 tool was used, with the following parameters: a mismatch cost of 3, an insertion cost of 3, a length fraction of 0.8, and a similarity fraction of 0.85.

### Cloning and heterologous expression of *IDI* proteins

Total RNA was extracted from frozen and ground *P. × canescens* leaves and *P. abies* needles using the InviTrap Spin Plant RNA Mini Kit (Invitek, Berlin, Germany), and cDNA libraries were prepared using the SuperScript III First-Strand Synthesis SuperMix (Thermo Fisher Scientific, Waltham, MA, USA) as previously described (Krause et al. 2023). For expression of IDI proteins, truncated sequences using the first methionine after the putative chloroplast transfer protein at position 86 for *Pa*IDI and 87 for *Pc*IDI (Figure S1a) were used for successive cloning into Gateway cloning™ (Thermo Fisher Scientific) vectors pDONR207 and pDEST15 using appropriate primers (Table S6). After transforming plasmids for IDI expression into BL21-AI™ One Shot™ Chemically Competent *E. coli* (Thermo Fisher Scientific), a 12 mL LB-preculture was directly inoculated and incubated for 72 h at 18 °C and 220 rpm. A 5 mL portion of the preculture was used to inoculate 100 mL of an LB overnight expression culture (Overnight Express™ Autoinduction System 1; Merck KGaA; Darmstadt, Germany), which was incubated for 24 h at 18 °C and 225 rpm. The whole procedure was performed with two technical replicates.

### Purification of the recombinant IDI protein

The induced culture was harvested by centrifugation at 4,000 × *g*, 4 °C for 20 min. The cell pellet was resuspended in 2 mL enzyme buffer (50 mM 2-hydroxy-3-morpholinopropanesulfonic acid (MOPSO), 10% (v/v) glycerol, 5 mM MgCl_2_, 200 mM KCl and 1 mM dithiothreitol (DTT), pH 7.0) and sonicated on ice for 5 min, 2 × pulse with 60% power using an UW2070 sonicator coupled to a Sonoplus HD2070 ultrasonic homogenizer (Bandelin Electronic, Berlin, Germany). The disrupted cell suspension was centrifuged for 20 min at 4 °C and 21,000 × *g,* and the supernatant was further purified using 3 mL Pierce™ Glutathione Spin Columns (Thermo Fisher Scientific) according to the manual. The binding of the enzyme to the column took place for 1 h at 4 °C in an end-over-end rotator. Protein concentration in the elution fractions was determined via a Bradford Assay using the Quick Start™ Bradford Protein Assay kit (Bio-Rad Laboratories GmbH, Hercules, CA, USA). A calibration curve was prepared with Bovine Serum Albumin (BSA) standards.

### IDI enzyme assays

A 2 × assay buffer consisted of a mixture of 100 mM Tris, 20% (v/v) glycerol, 10 mM MgCl_2_, 400 mM KCl, and 2 mM DTT (Phillips et al. 2008). The assays were performed in 300 µL portions with 150 µL 2 × assay buffer, 2 µg IDI protein, and variable amounts of dimethylallyl diphosphate (DMADP) or isopentenyl diphosphate (IDP), both as lithium salts. All compounds were purchased from Merck, Darmstadt, Germany. Assays were optimized regarding Zn^2+^ concentration, temperature and pH, testing conditions between 10 and 60 °C and 4.8 and 8.8, respectively. 40 °C and pH 7.0 were considered as optimal reaction conditions and applied in enzyme assays measuring kinetic parameters. All reactions were stopped by adding 200 µL chloroform and centrifuging for 5 min, at 4 °C and 11,000 × *g*. A 200 µL aliquot of the aqueous phase was transferred into new vials, mixed with 200 µL methanol, and stored at -80 °C until further analysis. Assays were performed in technical duplicates.

### Analysis of assay products and calculation of kinetic IDI parameters

For quantification of DMADP and IDP, analysis was performed using an Astec® Cyclobond® I 2,000 column (Krause et al. 2020). Initial velocities were calculated by plotting product formation over the different time points of the assays using Origin Pro (2019) followed by a fit using a BoxLucasI model (Equation: y = a · (1—exp(-b · x)) on the data set. Initial velocity was calculated by fitting a linear regression through y(0,0)=a*b. Data were transformed into Lineweaver-Burk-plots for visualization and Michaelis-Menten plots used to determine *K*_M_ and *k*_cat_.

### Determination of MEP pathway flux

MEP pathway flux was determined by following the incorporation of ^13^C label from ^13^CO_2_ into isoprene as described by Krause et al. (Krause et al. 2023). Briefly, plastidial concentrations of DXP, MEcDP, HMBDP, and IDP + DMADP (combined pool) were estimated from MS measurements, isoprene labeling was followed on-line instantaneously with proton transfer reaction-mass spectrometry, and the flux was estimated by mathematical fitting of the isoprene labeling time-course data.

### Vector construction and transformation of poplar and spruce

To target *PaIDI* mRNA for interference, the construction of the pCAMBIA1305.2 (www.cambia.org) vector was performed as described (Levée et al. 2009), and a fragment between nucleotide 88 and 275 of the coding sequence was selected. Transformation of *P. abies* embryogenic cell culture silencing *PaIDI* was carried out as described (Klimaszewska et al. 2001; Schmidt et al. 2010; Krause et al. 2023). To test the level of *PaIDI* expression, RT-qPCR analysis was done on WT, vector control (VC), and *PaIDI*-RNAi plants.

The transformation of the *P*. × *canescens* clone INRA 7171-B4 followed an established protocol (Meilan and Ma, 2006). For constructs overexpressing *PcIDI*, the complete open reading frame was cloned into pCAMGW, a gateway-compatible version of pCAMBIA2301, upstream of the maize (*Zea mays*) ubiquinone promoter promoter. To target *PcIDI* mRNA for interference, a fragment between nucleotide 89 and 275 of the coding sequence of *PcIDI* was selected and cloned in pCAMBIA1305.2, as it is described for *PaIDI*. The protocol has been described in detail (Schmidt et al. 2010; Krause et al. 2023). Transgenic RNAi plants were amplified by micropropagation as described by Behnke and coauthors (Behnke et al. 2007). Saplings of ∼10 cm high were repotted to soil (Klasmann potting substrate) and propagated in a controlled environment chamber for around 6 weeks (day, 22 °C; night, 18 °C; 65% relative humidity; 16 h/8 h light/dark cycle) before they were transferred to the greenhouse. To test the level of *PcIDI* expression, RT-qPCR analysis was done on WT, vector control (VC), RNAi-*PcIDI,* and OE-*PcIDI* plants.

The generation of transgenic *P*. × *canescens* that show silenced levels of *PcIDI* expression coupled to *PcHDR* overexpression followed a similar protocol. Transgenic plants containing the *PcIDI* silencing construct were transformed with a pCAMBIA1305.2 vector containing the *PcHDR*2 overexpression construct as described (Krause et al. 2023). After selection for the correct transformants, plants were treated as described above and later screened for the levels of *PcIDI* and *PcHDR*2 expression using RT-qPCR analysis.

### Analysis of poplar plants growing on IDP or isoprenol-enriched medium

Poplar saplings (*P*. × *canescens*) were grown on solid half strength MS medium in Magenta™ vessels (SIGMA) until they reached a height of approximately 4 cm. Plants were transferred into 850 mL glass jars (WECK, Wehr-Öflingen, Germany), filled with 100 mL solid half strength MS medium containing 10 or 30 µM isopentenyl pyrophosphate triammonium salt dissolved (Merck, Darmstadt, Germany) in MeOH/10 mM NH_4_OH (7:3), 0.2 or 0.5 M isoprenol (Merck, Darmstadt, Germany), or mock controls with MeOH/10 mM NH_4_OH (7:3) or MeOH. Three plants per jar were used as biological replicates. After 7, 14, and 21 days, volatiles were trapped for 24 h using preconditioned 5 mm long polydimethysiloxane (PDMS) tubes that were attached to the lid using a curved needle. Three PDMS tubes per measurement were used. Isoprenol and isoprenyl acetate were analyzed on a TD-20 thermal adsorption unit coupled to a GCMS-QP2010 system (Shimadzu) as described below. Afterwards, leaves were harvested for prenyl diphosphate analysis and RNA extraction. Leaves were detached and weighed (fresh weight), and plant material from each jar was combined and further processed. The emission of isoprenol and isoprenyl acetate and the content of IDP were all expressed in relative terms and normalized by tissue weight to compare the results of volatile collection and tissue extraction.

### RNA-seq and quantitative reverse transcription polymerase chain reaction (RT-qPCR) analysis

Total RNA was extracted from frozen plant material as described above. RNA from wild type *P.* × *canescens* plants was subjected to RNA-seq analysis, which was performed by Novogene (Beijing, China). Trimmed reads were mapped against the *P. trichocarpa* genome using HISAT2 software, and RPKM values were calculated.

For gene expression analysis of the *IDI* genes of poplar and spruce, as well as *PcHDR*1 and 2, and *PcIS*, cDNA was prepared as described above, and primers were used as described (Krause et al. 2023). Ubiquitin was used as a reference for relative quantification of expression. Each biological replicate was measured with three technical duplicates.

### Collection and analysis of isoprene and other volatile terpenoids

For analyzing isoprene emission in spruce, young saplings with a size of approximately 15 cm were placed in 3 L glass desiccator and equilibrated for several hours. After closing the lid, the jar was attached to a push-pull-system with air pumped into and out of the desiccator at a constant flow of 1 L × min^−1^. Charcoal filtered air was used and volatiles were trapped using fitted thermal desorption tubes filled with 60 mg Carbotrap® X, 20 to 40 mesh (Merck KGaA, Darmstadt, Germany) for 10 min. Samples were analyzed by gas chromatography coupled to mass spectrometry (GC-MS) analysis. After headspace collection, needles were immediately harvested, weighed and extracted for other metabolites as described below. For poplar, headspace volatiles were collected and analyzed as described (Krause et al. 2023). In short, plants were enclosed with PET bags (“Bratschlauch”, Toppits, Minden, Germany) with the ends sealed (McCormick et al. 2014). Isoprene and terpenoid emission were collected over 24 h, trapped using glass tubes filled with 20 mg Porapak-Q™ (http://www.volatilecollectiontrap.com) and eluted with 200 µL dichloromethane. Qualitative and quantitative analysis of the samples was conducted using GC-MS or FID analysis.

### Analysis of other terpenoids

Monoterpenoid, sesquiterpenoid, and diterpenoid compounds were extracted together from spruce needles and analyzed as described (Krause et al. 2023). Monoterpenes and sesquiterpenes were analyzed directly by GC-MS, while diterpene acids were first methylated before GC-MS analysis. Carotenoids and chlorophylls from foliage of both species were extracted and analyzed using HPLC with UV detection; sterols from poplar leaves were extracted and analyzed using GC-MS measurements. Both methods are described by Nagel et al. (2014).

### DMADP, IDP, and short-chain prenyl diphosphate analysis

DMADP and IDP were extracted from poplar leaves and spruce needles and analyzed by LC-MS with a β-cyclodextrin column as described (Krause et al. 2020). GDP, FDP, and GGDP were analyzed by triple quadrupole LC-MS (Nagel et al. 2014). Multiple reactions monitoring (MRM) was used to monitor analyte parent-ion-to-product-ion formation, and the machine was operated in negative ionization mode. Dimethylallyl-S-thiolodiphosphate (DMASP) served as the internal standard.

### Statistical analysis

Statistical analysis for transgenic lines was performed by comparing them with empty vector controls using Student's t-tests. Tissue specific *Pa* and *PcIDI* gene expression, and experiments with poplar saplings supplemented with IDP were analyzed using ANOVA. Significance is shown by asterisks, representing *P*-values of ≤0.05, ≤0.01, and ≤0.001 with *, **, and ***, respectively.

### Accession numbers

Sequence data from this article can be found in the GenBank/NCBI or PlantGenIE data libraries under the accession numbers listed in the “Materials and Methods’ section, in the chapter “IDI sequences from poplar and spruce’ subsection.

## Supplementary Material

kiag225_Supplementary_Data

## Data Availability

The author responsible for the distribution of materials integral to the findings presented in this article, in accordance with the policy described in the Instructions for Authors (https://academic.oup.com/plphys/pages/general-instructions) is: Axel Schmidt (aschmidt@ice.mpg.de).
