# Supplementary material for: Isopentenyl diphosphate isomerase exerts limited control over terpenoid biosynthesis in two woody plant species
Source: Plant Physiol. 2026 Apr 21;201(1):kiag225. doi: 10.1093/plphys/kiag225 (PMC13222028; doi:10.1093/plphys/kiag225)
Supplement: kiag225_Supplementary_Data [file kiag225_supplementary_data.pdf]

Isopentenyl diphosphate isomerase exerts limited control over terpenoid biosynthesis in two woody plant species

Toni Krause, Kristina Kshatriya, Jia Zhang, Johann M. Rohwer, Jonathan Gershenzon and Axel Schmidt

Corresponding author:

Axel Schmidt

Email: aschmidt@ice.mpg.de

This file includes:

Figures S1 to S9

Tables S1 to S11

A

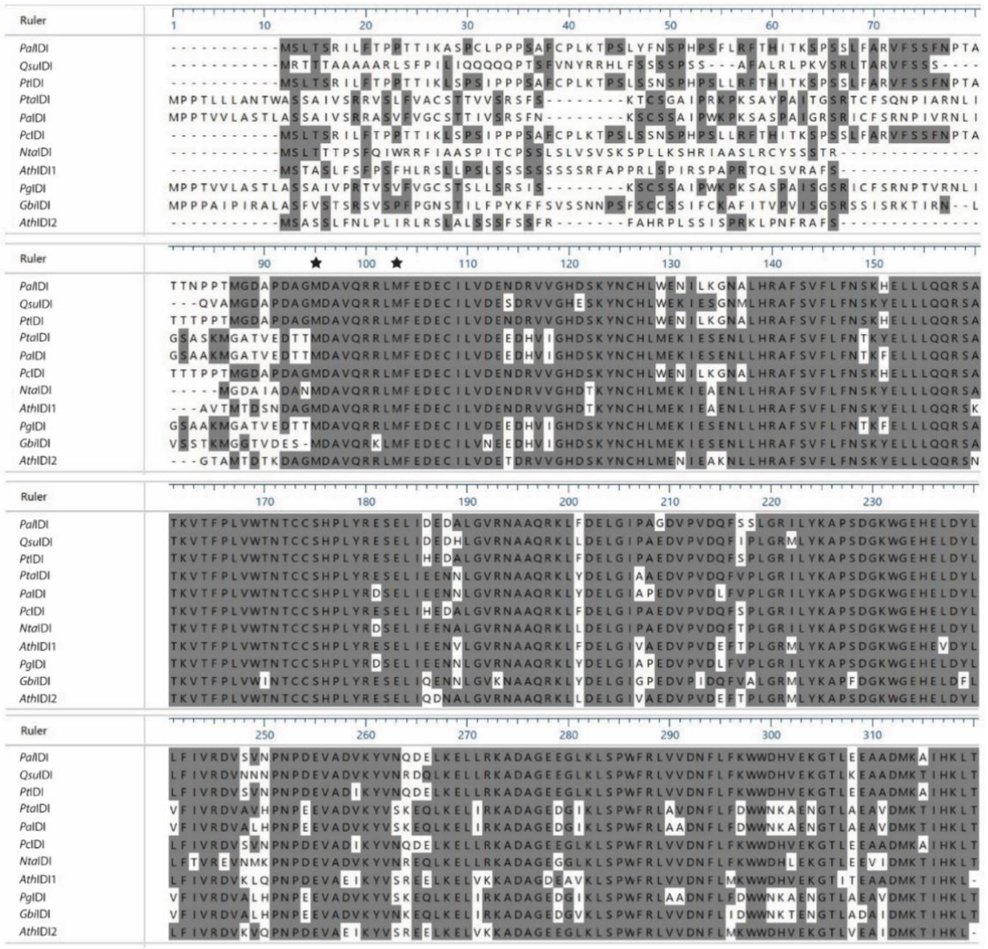

B

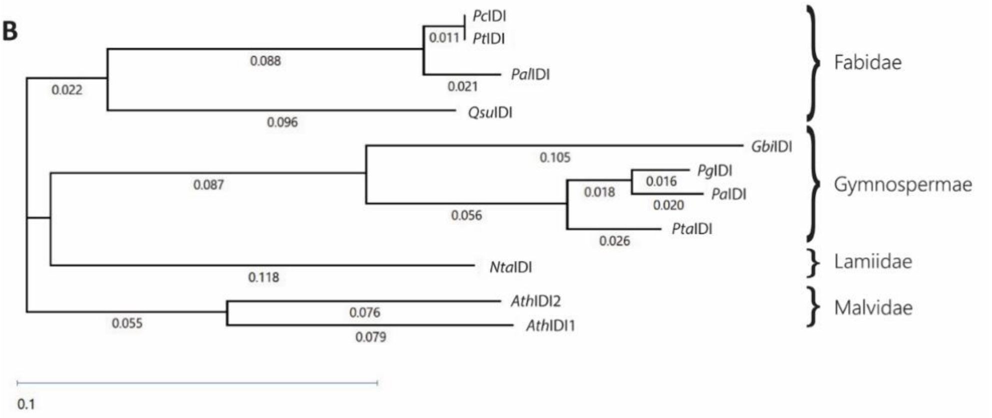

C

Program	*Pa* IDI-M1	*Pa* IDI-M2	*Pc* IDI-M1	*Pc* IDI-M2
**iPSORT**	Signal peptide	NA	Plastid	NA
**Predotar**	Plastid	NA	Plastid	NA
**TargetP**	Plastid 0.6850	NA	Plastid 0.9361	NA
**PTS1 predictor**	NA	NA	NA	NA
**WoLF PSORT**	Plastid	Plastid	Plastid	Cytoplasm
**DeepLoc 2.1**	Plastid 0.8518 Mitochondrial 0.6373	Cytoplasm 0.7686	Plastid 0.9237	Peroxisome 0.7685 Cytoplasm 0.7459

Supplementary Figure S1. Phylogenetic analysis of amino acid sequences of IDIs from poplar, spruce and other plant species. Sequences were obtained from the NCBI database and aligned with the MegAlign Pro ClustalW algorithm. The unit of the branch length is substitutions per site. Sequences with their Gene Bank accession numbers are *Pc*IDI (*P. × canescens* IDI; identical to *Pt*IDI), *Pt*IDI (*Populus trichocarpa* IDI; XP_002325469.2), *Pal*IDI (*Populus alba* IDI; XP_034888506.1), *Qsu*IDI (*Quercus suber* IDI; XP_023892310.1), *Gbi*IDI (*Gingko biloba* IDI; ACU56979.1), *Pg*IDI (*Picea glauca* IDI; BT108967.1), *Pa*IDI (*Picea abies* IDI; PQ581929), *Pta*IDI (*Pinus taeda* IDI; QBS32937.1), *Nta*IDI (*Arabidopsis thaliana* IDI; NP_001313140.1), *Ath*IDI1 (*A. thaliana* IDI1; NP_197148.3), *Ath*IDI2 (*A. thaliana* IDI1; NP_186927). All proteins contain a putative plastid-targeting peptide (determined with TargetP 2.0; DTU Health Tech; Denmark). Two methionine residues that might represent alternative initial amino acids of the protein are indicated by stars **A**). Phylogenetic relationships of the sequences **B**). Bioinformatic predictions for subcellular localization of *Pa*IDI and *Pc*IDI protein versions; M1 corresponds to the first methionine of the ORF, M2 to the second methionine of the ORF, at position 86 for *Pa* or 87 for *Pc* (Supplementary Fig. S1A). NA, Not applicable, indicates that program did not have the capacity to make a prediction for this sequence **C**).

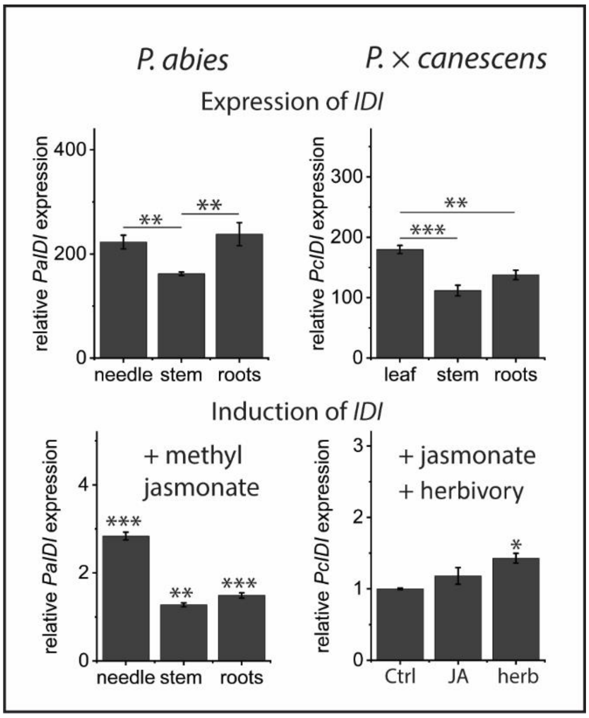

Supplementary Figure S2. Isopentenyl diphosphate isomerase (IDI) gene expression in spruce and poplar. qRT-PCR analysis of different organs from wild-type spruce and poplar plants revealed specific expression patterns of IDI. Treating spruce with methyl jasmonate (MJ) caused significant induction of *PaIDI* gene expression. In poplar, this effect was also observed in leaves after herbivory by *Chrysomela populi* leaf beetles (herbivory), but not after jasmonic acid (JA) treatment. Values in the induction experiments (lower panels) were normalized to organ-specific controls without treatment. Values are given as means ± standard deviation of three biological replicates, measured as technical duplicates. *** = $p < 0.001$; ** = $p < 0.01$; * = $p < 0.05$; ANOVA. For *p*-values see Supplementary Table S7.

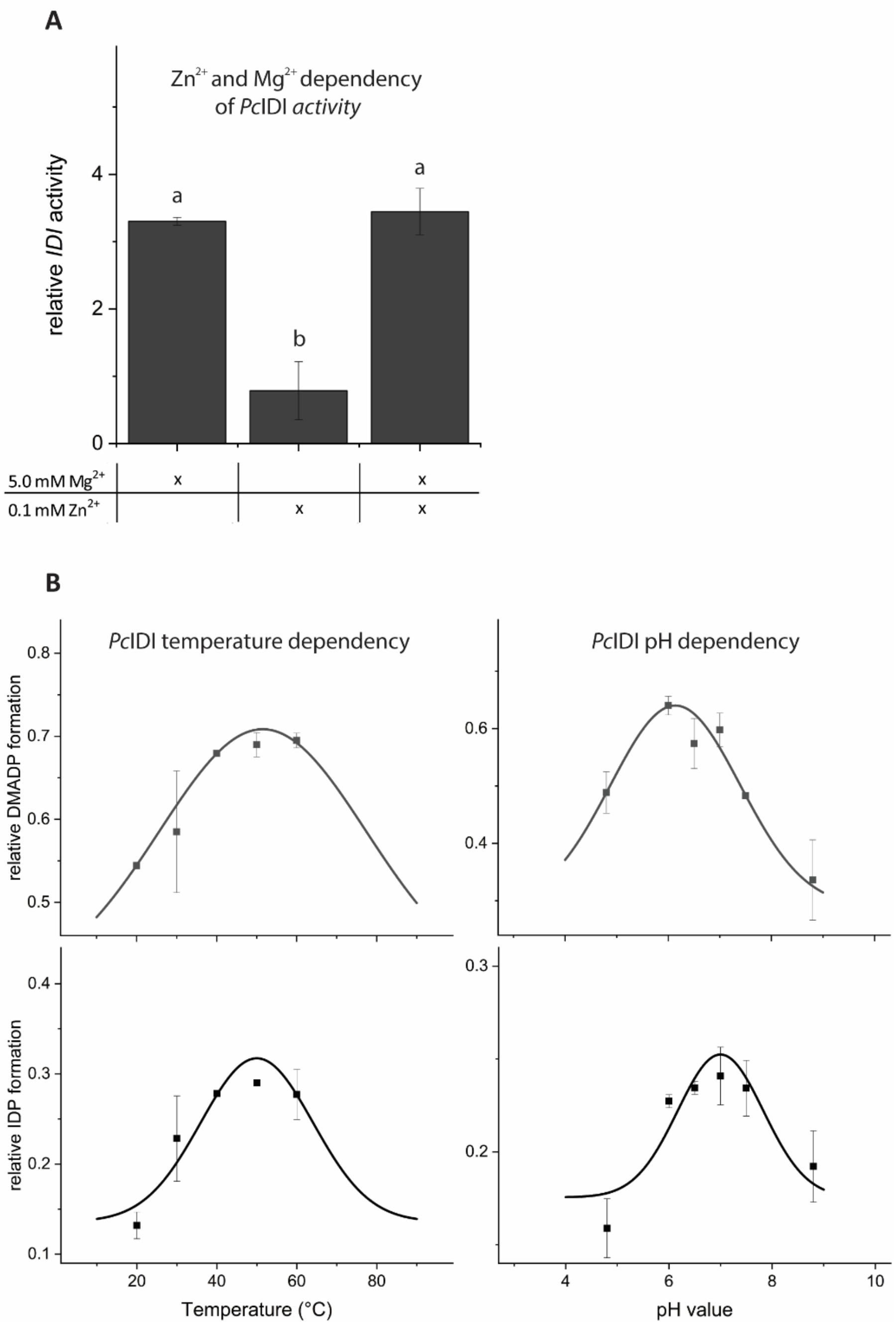

Supplementary Figure S3. Heterologously expressed recombinant IDI of *Populus* × *canescens* was assayed under different metal ion, pH and temperature conditions. Recombinant IDI was heterologously expressed in *E. coli* and tested for its ability to catalyze the isomerization of dimethylallyl diphosphate (DMADP) to isopentenyl diphosphate (IDP) and *vice versa*. Dependency of IDI activity on different metal ion combinations **A**). Dependency of activity on temperature and pH conditions **B**). Each data point represents the mean ± standard deviation of two biological replicates, measured in technical duplicates. *** = p < 0.001; Student's t-test. For *p*-values see Supplementary Table S8.

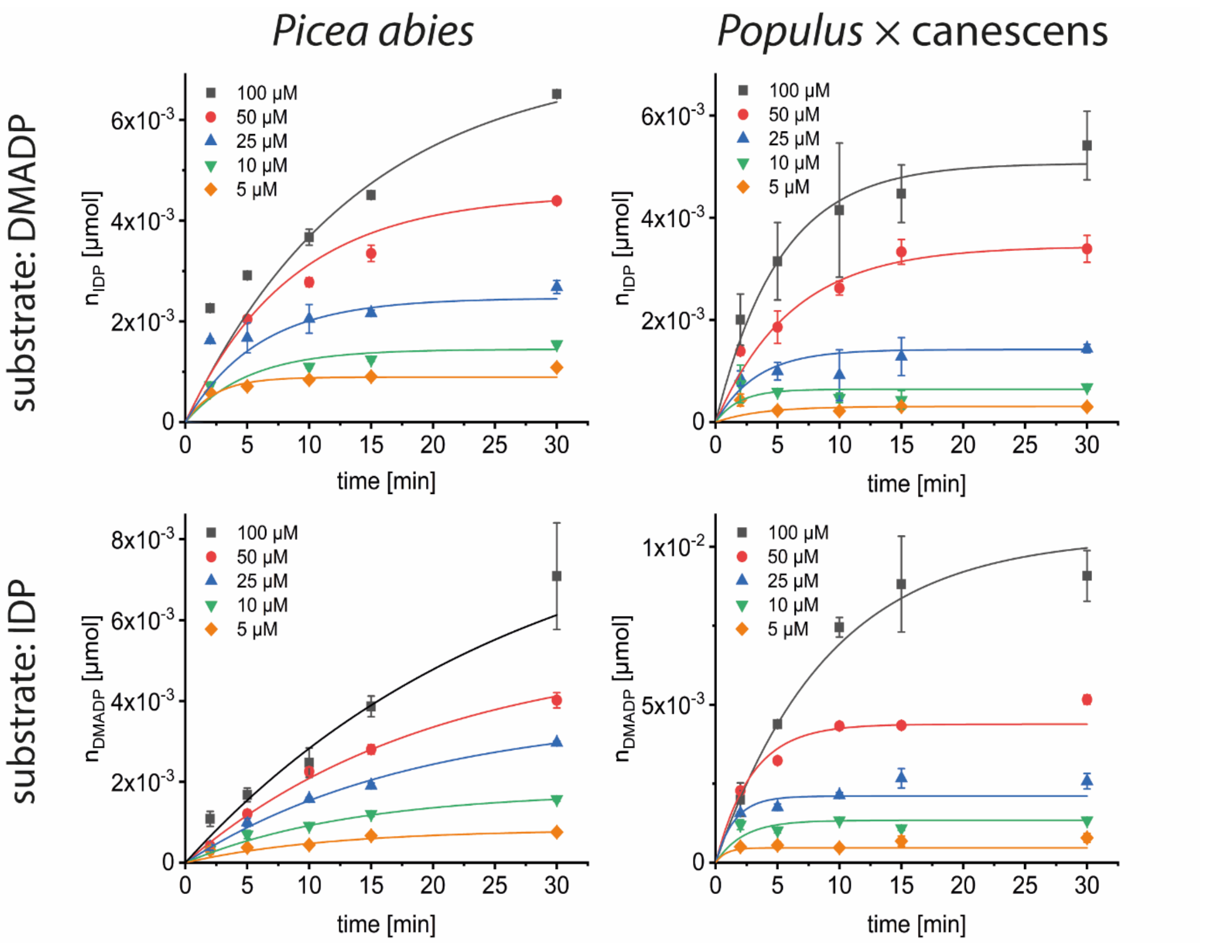

Supplementary Figure S4. Initial velocity plots of spruce and poplar IDI enzymes using DMADP and IDP as substrates. Several different concentrations of DMADP or IDP were tested for their conversion rates using heterologously expressed and purified IDI from spruce and poplar. Using an exponential fitting model, initial velocities were calculated. Each data point represents the mean ± standard deviation of two biological replicates, measured in technical duplicates.

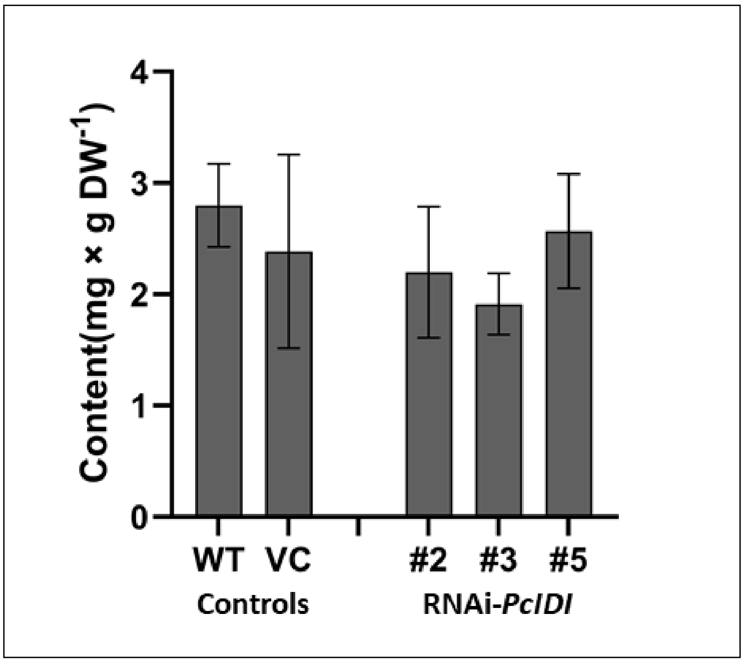

Supplementary Figure S5. Content of β-sitosterol in transgenic *Populus* × *canescens* lines and controls. β-sitosterol was analyzed by GC-MS measurements. *PcIDI* silencing had no significant effect on the content of β-sitosterol in leaves. Values are given as mean ± standard deviation of at least three biological replicates per line. Statistical analyses comparing transgenic lines with controls were performed using One-way ANOVA. WT, wild-type control; VC, vector controls. For p-values see Supplementary Table S9.

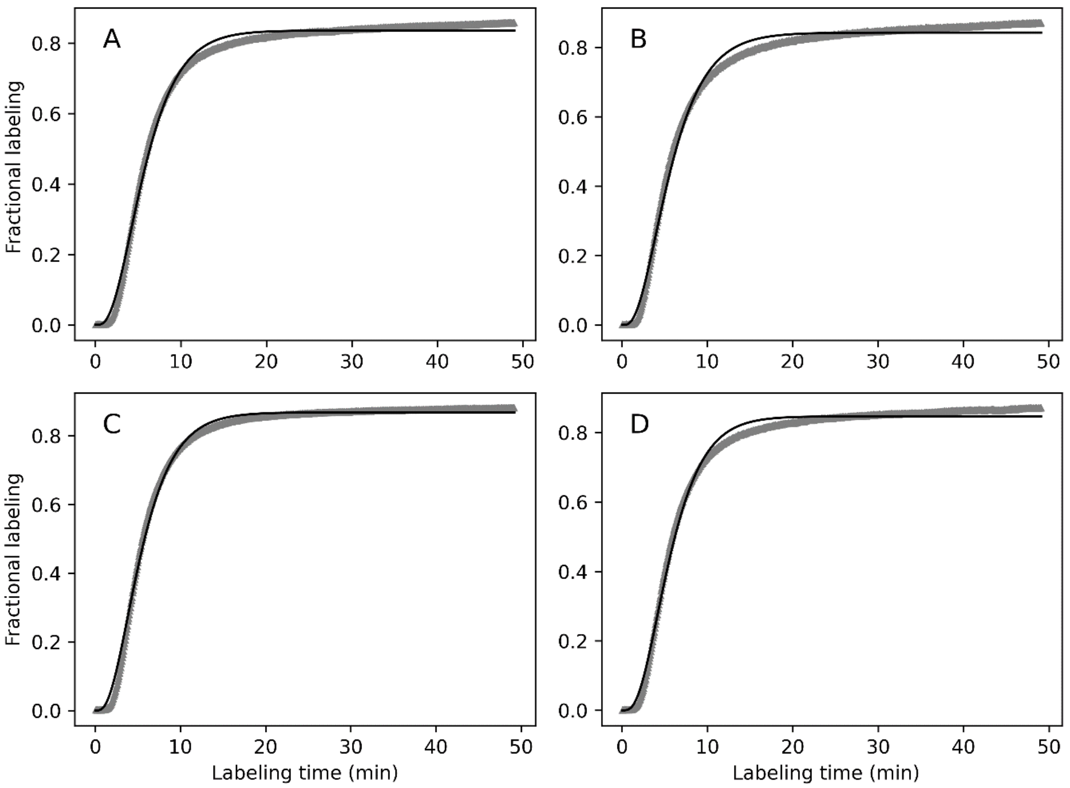

Supplementary Figure S6. Determining MEP pathway flux from fitting to time-courses of ^{13}C label incorporation into isoprene. Shown are representative samples of fractional labeling of isoprene (grey symbols) as measured with PTR-MS for *PcIDI*-silenced lines #2 (A), #3 (B), #5 (C) and #8 (D). Black lines indicate the fitted mathematical model used to calculate the flux (see main text, Materials and Methods).

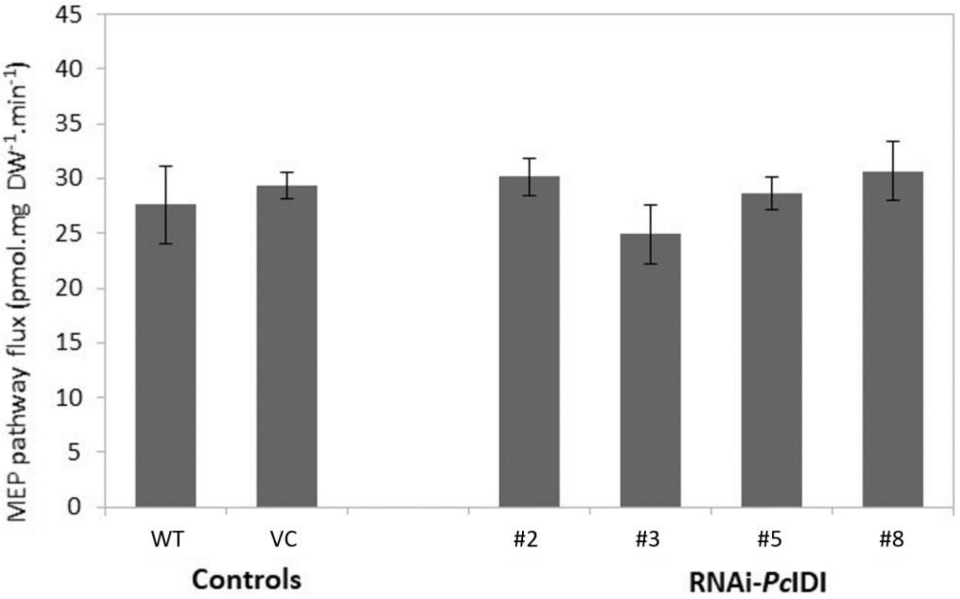

Supplementary Figure S7. Effects of *PcIDI* gene silencing on methylerythritol phosphate (MEP) pathway flux in transgenic *Populus* × *canescens* lines. Flux was determined from fitting of time-courses of label incorporation from $^{13}CO_2$ into isoprene, as shown in Supplementary Figure S6. *PcIDI* silencing had no significant effect on the flux through the pathway. Values are given as mean ± standard error of 3 – 5 biological replicates per line. Statistical analysis was performed by using Student's t-test of individual lines vs. vector controls. For *p*-values see Supplementary Table S10.

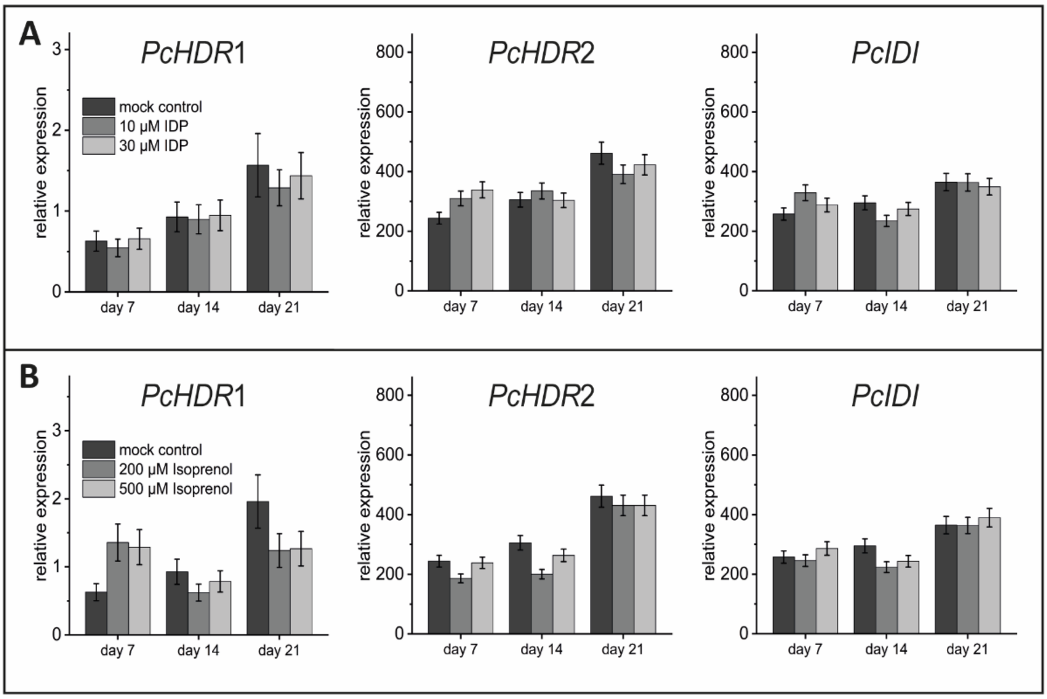

Supplementary Figure S8. RT-qPCR analysis of *PcHDR*1, *PcHDR*2 and *PcIDI* from young poplar saplings supplemented with isopentenyl diphosphate (IDP) or isoprenol. Three plants per pot were grown and supplemented with IDP **A)** or isoprenol **B)** and leaves harvested after 7, 14 and 21 days. Plants were pooled and RNA was extracted for analysis. Values represent mean ± standard deviation of three biological replicates, measured in technical duplicates. Expression did not change significantly when saplings were supplemented with IDP or isoprenol. ANOVA. For *p*-values see Supplementary Table S11.

	Norway spruce (*P. abies*) RNAi *Pa*IDI	Gray Poplar (*Populus × canescens*) RNAi *Pc*IDI	Gray Poplar (*Populus × canescens*) RNAi *Pc*IDI/OE-*Pc*HDR
Dimethylallyl diphosphate	-	-	-
Isopentenyl diphosphate	↑	↑	↑
Isoprene	-	↓	-
Geranyl diphosphate	↓	-	↑
Farnesyl diphosphate	-	↑	-
Geranylgeranyl diphosphate	-	↑	↑
Monoterpenes	↓	-	NA
Sesquiterpenes	↓	-	NA
Diterpenes	-	ND	NA
Carotenoids	-	-	↑
Chlorophyll	-	-	↑
Sterols	NA	-	NA
Isoprenol	↑	↑	↑
Isoprenyl acetate	↑↑	↑↑	↑↑

Supplementary Figure S9. Summarizing scheme linking the results from both species. Dashes (-) indicate no change in metabolite concentrations, while arrows (↑, ↑↑, ↓) indicate upregulation, strong upregulation, or downregulation.NA - not applicable, ND - not detectable

Supplementary Table S1. *p*-values for Fig. 3

		**Student's t-test**
**Interaction**	**Sample**	***p*-value**
Expression	RNAi-*PaIDI*#14 × VC	<0.001
Expression	RNAi-*PaIDI*#17 × VC	<0.001
DMADP	RNAi-*PaIDI*#14 × VC	0.941
DMADP	RNAi-*PaIDI*#17 × VC	0.372
IDP	RNAi-*PaIDI*#14 × VC	<0.001
IDP	RNAi-*PaIDI*#17 × VC	<0.001
DMADP : IDP	RNAi-*PaIDI*#14 × VC	0.006
DMADP : IDP	RNAi-*PaIDI*#17 × VC	<0.001
Isoprene	RNAi-*PaIDI*#14 × VC	0.494
Isoprene	RNAi-*PaIDI*#17 × VC	0.069
GDP	RNAi-*PaIDI*#14 × VC	0.080
GDP	RNAi-*PaIDI*#17 × VC	0.006
Monoterpenes	RNAi-*PaIDI*#14 × VC	<0.001
Monoterpenes	RNAi-*PaIDI*#17 × VC	<0.001
FDP	RNAi-*PaIDI*#14 × VC	0.972
FDP	RNAi-*PaIDI*#17 × VC	0.210
Sesquiterpenes	RNAi-*PaIDI*#14 × VC	0.002
Sesquiterpenes	RNAi-*PaIDI*#17 × VC	0.005
GGDP	RNAi-*PaIDI*#14 × VC	0.795
GGDP	RNAi-*PaIDI*#17 × VC	0.918
Carotenoids	RNAi-*PaIDI*#14 × VC	0.431
Carotenoids	RNAi-*PaIDI*#17 × VC	0.207
Chlorophylls	RNAi-*PaIDI*#14 × VC	0.878
Chlorophylls	RNAi-*PaIDI*#17 × VC	0.216
Diterpenes	RNAi-*PaIDI*#14 × VC	0.660
Diterpenes	RNAi-*PaIDI*#17 × VC	0.990

Supplementary Table S2. *p*-values for Fig. 4

		**Student's t-test**
**Interaction**	**Sample**	***p*-value**
Expression	OE-*PcIDI*#2 × VC	<0.001
Expression	OE-*PcIDI*#20 × VC	<0.001
Expression	OE-*PcIDI*#25 × VC	<0.001
Expression	RNAi-*PcIDI*#2 × VC	<0.001
Expression	RNAi-*PcIDI*#3 × VC	<0.001
Expression	RNAi-*PcIDI*#5 × VC	<0.001
DMADP	OE-*PcIDI*#2 × VC	0.120
DMADP	OE-*PcIDI*#20 × VC	0.524
DMADP	OE-*PcIDI*#25 × VC	0.221
DMADP	RNAi-*PcIDI*#2 × VC	0.096
DMADP	RNAi-*PcIDI*#3 × VC	0.147
DMADP	RNAi-*PcIDI*#5 × VC	0.061
IDP	OE-*PcIDI*#2 × VC	0.382
IDP	OE-*PcIDI*#20 × VC	0.467
IDP	OE-*PcIDI*#25 × VC	0.888
IDP	RNAi-*PcIDI*#2 × VC	<0.001
IDP	RNAi-*PcIDI*#3 × VC	<0.001
IDP	RNAi-*PcIDI*#5 × VC	<0.001
DMADP : IDP	OE-*PcIDI*#2 × VC	<0.001
DMADP : IDP	OE-*PcIDI*#20 × VC	0.010
DMADP : IDP	OE-*PcIDI*#25 × VC	0.020
DMADP : IDP	RNAi-*PcIDI*#2 × VC	<0.001
DMADP : IDP	RNAi-*PcIDI*#3 × VC	<0.001
DMADP : IDP	RNAi-*PcIDI*#5 × VC	0.004
GDP	OE-*PcIDI*#2 × VC	0.083
GDP	OE-*PcIDI*#20 × VC	0.517
GDP	OE-*PcIDI*#25 × VC	0.068
GDP	RNAi-*PcIDI*#2 × VC	0.092
GDP	RNAi-*PcIDI*#3 × VC	0.189
GDP	RNAi-*PcIDI*#5 × VC	0.186
FDP	OE-*PcIDI*#2 × VC	0.474
FDP	OE-*PcIDI*#20 × VC	0.964
FDP	OE-*PcIDI*#25 × VC	0.187
FDP	RNAi-*PcIDI*#2 × VC	0.003
FDP	RNAi-*PcIDI*#3 × VC	0.002
FDP	RNAi-*PcIDI*#5 × VC	0.002
GGDP	OE-*PcIDI*#2 × VC	0.211
GGDP	OE-*PcIDI*#20 × VC	0.124
GGDP	OE-*PcIDI*#25 × VC	0.290
GGDP	RNAi-*PcIDI*#2 × VC	0.009
GGDP	RNAi-*PcIDI*#3 × VC	0.019

GGDP	RNAi-*PcIDI*#5 × VC	0.005
Monoterpenes	OE-*PcIDI*#2 × VC	0.926
Monoterpenes	OE-*PcIDI*#20 × VC	0.575
Monoterpenes	OE-*PcIDI*#25 × VC	0.361
Monoterpenes	RNAi-*PcIDI*#2 × VC	0.342
Monoterpenes	RNAi-*PcIDI*#3 × VC	0.435
Monoterpenes	RNAi-*PcIDI*#5 × VC	0.823
Sesquiterpenes	OE-*PcIDI*#2 × VC	0.804
Sesquiterpenes	OE-*PcIDI*#20 × VC	0.958
Sesquiterpenes	OE-*PcIDI*#25 × VC	0.266
Sesquiterpenes	RNAi-*PcIDI*#2 × VC	0.116
Sesquiterpenes	RNAi-*PcIDI*#3 × VC	0.612
Sesquiterpenes	RNAi-*PcIDI*#5 × VC	0.495
Caro/Chloro	OE-*PcIDI*#2 × VC	0.700
Caro/Chloro	OE-*PcIDI*#20 × VC	0.898
Caro/Chloro	OE-*PcIDI*#25 × VC	0.062
Caro/Chloro	RNAi-*PcIDI*#2 × VC	0.760
Caro/Chloro	RNAi-*PcIDI*#3 × VC	0.968
Caro/Chloro	RNAi-*PcIDI*#5 × VC	0.161

Supplementary Table S3. *p*-values for Fig. 5

		Student's t-test
**Interaction**	**Sample**	***p*-value**
Expression IS	OE-*PcIDI*#2 × VC	0.737
Expression IS	OE-*PcIDI*#20 × VC	0.702
Expression IS	OE-*PcIDI*#25 × VC	0.767
Expression IS	RNAi-*PcIDI*#2 × VC	0.811
Expression IS	RNAi-*PcIDI*#3 × VC	0.340
Expression IS	RNAi-*PcIDI*#5 × VC	0.330
Isoprenol	OE-*PcIDI*#2 × VC	0.159
Isoprenol	OE-*PcIDI*#20 × VC	0.811
Isoprenol	OE-*PcIDI*#25 × VC	0.951
Isoprenol	RNAi-*PcIDI*#2 × VC	<0.001
Isoprenol	RNAi-*PcIDI*#3 × VC	<0.001
Isoprenol	RNAi-*PcIDI*#5 × VC	<0.001
Isoprene	OE-*PcIDI*#2 × VC	0.760
Isoprene	OE-*PcIDI*#20 × VC	0.981
Isoprene	OE-*PcIDI*#25 × VC	0.967
Isoprene	RNAi-*PcIDI*#2 × VC	<0.001
Isoprene	RNAi-*PcIDI*#3 × VC	<0.001
Isoprene	RNAi-*PcIDI*#5 × VC	<0.001
Isoprenyl acetate	OE-*PcIDI*#2 × VC	0.473
Isoprenyl acetate	OE-*PcIDI*#20 × VC	0.885
Isoprenyl acetate	OE-*PcIDI*#25 × VC	0.606
Isoprenyl acetate	RNAi-*PcIDI*#2 × VC	<0.001
Isoprenyl acetate	RNAi-*PcIDI*#3 × VC	<0.001
Isoprenyl acetate	RNAi-*PcIDI*#5 × VC	<0.001
Isoprenol	RNAi-*PaIDI*#14 × VC	0.031
Isoprenol	RNAi-*PaIDI*#17 × VC	<0.001
Isoprenyl acetate	RNAi-*PaIDI*#14 × VC	0.089
Isoprenyl acetate	RNAi-*PaIDI*#17 × VC	0.002

Supplementary Table S4. Gene expression, terpene content, and emissions in double transgenic (RNAi-*PcIDI*/OE-*PcHDR*) lines compared to vector controls (VC) and single *PcIDI*-knock-down lines (RNAi-*PcIDI*) (Fig. 6) - statistical analysis and *p*-values

	Target	unit	RNAi-*PcIDI*/OE-*PcHDR* #1 or #2			
			VC vs #1	VC vs #3	RNAi-*PcIDI* vs #1	RNAi-*PcIDI* vs #3
gene expression	*PcIDI*	ddCq × 1000	<0.001	<0.001	0.936	0.403
	PcHDR2		<0.001	0.001	0.007	0.008
	PcIS		<0.001	0.012	0.016	0.014
content	DMADP	ng × mg FW^{-1}	0.967	0.592	0.712	0.505
	IDP		<0.001	<0.001	<0.001	0.007
	GDP		0.020	0.010	0.376	0.349
	FDP		0.650	0.020	<0.001	0.763
	GGDP		<0.001	<0.001	0.006	0.211
	Carotenoids		0.013	0.029	<0.001	<0.001
	Chlorophylls		0.009	0.015	<0.001	0.005
emission	Isoprene	ng × g FW^{-1} x day^{-1}	0.052	0.263	0.409	0.225
	Isoprenol		<0.001	<0.001	0.005	0.002
	Isoprenyl acetate		<0.001	<0.001	0.020	0.029

Transgenic poplar with silenced *PcIDI* gene expression and overexpression of *PcHDR* (RNAi-*PcIDI*/OE-*PcHDR*) were compared with vector controls (VC) and transgenic poplar with exclusively silenced *PcIDI* gene expression (RNAi-*PcIDI*). Analysis was performed using Student's t-test and *p*-values related to Fig. 6 are shown.

Supplementary Table S5. *p*-values for Fig. 7

		ANOVA
**Interaction**	**Sample**	***p*-value**
IDP / + IDP	day 7 10 uM × mock	<0.001
IDP / + IDP	day 7 30 uM × mock	<0.001
IDP / + IDP	day 14 10 uM × mock	0.071
IDP / + IDP	day 14 30 uM × mock	<0.001
IDP / + IDP	day 21 10 uM × mock	0.082
IDP / + IDP	day 21 30 uM × mock	<0.001
IDP / + Isoprenol	day 7 10 uM × mock	0.143
IDP / + Isoprenol	day 7 30 uM × mock	0.268
IDP / + Isoprenol	day 14 10 uM × mock	0.089
IDP / + Isoprenol	day 14 30 uM × mock	0.166
IDP / + Isoprenol	day 21 10 uM × mock	0.257
IDP / + Isoprenol	day 21 30 uM × mock	0.189
Isoprenol / + IDP	day 7 10 uM × mock	<0.001
Isoprenol / + IDP	day 7 30 uM × mock	<0.001
Isoprenol / + IDP	day 14 10 uM × mock	<0.001
Isoprenol / + IDP	day 14 30 uM × mock	<0.001
Isoprenol / + IDP	day 21 10 uM × mock	0.041
Isoprenol / + IDP	day 21 30 uM × mock	<0.001
Isoprenol / + Isoprenol	day 7 10 uM × mock	<0.001
Isoprenol / + Isoprenol	day 7 30 uM × mock	<0.001
Isoprenol / + Isoprenol	day 14 10 uM × mock	<0.001
Isoprenol / + Isoprenol	day 14 30 uM × mock	<0.001
Isoprenol / + Isoprenol	day 21 10 uM × mock	<0.001
Isoprenol / + Isoprenol	day 21 30 uM × mock	<0.001
Isoprenyl acetate/ + IDP	day 7 10 uM × mock	0.324
Isoprenyl acetate/ + IDP	day 7 30 uM × mock	<0.001
Isoprenyl acetate/ + IDP	day 14 10 uM × mock	0.033
Isoprenyl acetate/ + IDP	day 14 30 uM × mock	<0.001
Isoprenyl acetate/ + IDP	day 21 10 uM × mock	0.042
Isoprenyl acetate/ + IDP	day 21 30 uM × mock	<0.001
Isoprenyl acetate/ + Isoprenol	day 7 10 uM × mock	<0.001
Isoprenyl acetate/ + Isoprenol	day 7 30 uM × mock	<0.001
Isoprenyl acetate/ + Isoprenol	day 14 10 uM × mock	<0.001
Isoprenyl acetate/ + Isoprenol	day 14 30 uM × mock	<0.001
Isoprenyl acetate/ + Isoprenol	day 21 10 uM × mock	<0.001
Isoprenyl acetate/ + Isoprenol	day 21 30 uM × mock	<0.001

Supplementary Table S6. Primers used in this study.

Name	Sequence 5’-3’	usage
PcIDI-T_GW_fwd	GGGGACAAGTTTGTACAAAAAAGCAGGCTTCAT GGGTGACGCTCCTGATG	Hetero exp. in *E. coli*
PcIDI_GW_rev	GGGGACCACTTTGTACAAGAAAGCTGGGTCTCA AGTCAGCTTGTGAATCGCTTTC	Hetero exp. in *E. coli*
PaIDI-T_GW_fwd	GGGGACAAGTTTGTACAAAAAAGCAGGCTTCAT GGGGGCGACAGTGGAG	Hetero exp. in *E. coli*
PaIDI_GW_rev	GGGGACCACTTTGTACAAGAAAGCTGGGTCTCA AGTCAACTTATGTATGGTTTTCATATC	Hetero exp. in *E. coli*
PcIDI_qPCR_fwd	ACGTCAAGTACGTTAACCAGGA	RT-qPCR
PcIDI_qPCR_rev	TGGTCCCACCACTTGAACAG	RT-qPCR
PcHDR1_qPCR_fwd	CGCCGTATAACCACCGTGT	RT-qPCR
PcHDR1_qPCR_rev	TATGTCTGAACACTTTGGCGTC	RT-qPCR
PcHDR2_qPCR_rev	AGGTATAATCTCCCTTCTTGTGCT	RT-qPCR
PcIS_qPCR_fwd	ACACACAAACTGTTCAGAAATCCC	RT-qPCR
PcIS_qPCR_rev	CCGTCTGGCTTCTGTTTCTGT	RT-qPCR
PcUbi_qPCR_fwd	GTTGATTTTTGCTGGGAAGC	RT-qPCR
PcUbi_qPCR_rev	GATCTTGGCCTTCACGTTGT	RT-qPCR
PaIDI_qPCR_fwd	TGGAGGATACGACCATGGATG	RT-qPCR
PaIDI_qPCR_rev	TCATGCCCAATGACATGATCTTC	RT-qPCR
PaUbi_qPCR_fwd	GTTGATTTTTGCTGGCAAGC	RT-qPCR
PaUbi_qPCR_rev	CACCTCTCAGACGAAGTAC	RT-qPCR
*PcIDI*_OE_fwd	GGGGACAAGTTTGTACAAAAAAGCAGGCTTCA CCATGTCTCTGACCTCTCGCATTC	Poplar
*PcIDI*_OE_rev	GGGGACCACTTTGTACAAGAAAGCTGGGTCTCA AGTCAGCTTGTGAATCGCTTTC	Poplar
*PcIDI*_RNAi_fwd	TGCTCTAGAGCAGGGTTGTTGGTCATGACTCC	Poplar
*PcIDI*_RNAi _rev	CGGGATCCCGGGTAGGCTGCAGCAAGTGATA	Poplar
*PaIDI*_RNAi_fwd	TGCTCTAGAGCAGATCATGTCATTGGGCATGAC	Spruce
*PaIDI*_RNAi _rev	CGGGATCCCGGGATGGCTGCAGCAGGTATTT	Spruce

Supplementary Table S7. *p*-values for Supplementary Fig. S2

		ANOVA
**Interaction**	**Sample**	***p*-value**
Expression	*PaIDI* needle × *PaIDI* stem	0.008
Expression	*PaIDI* roots × *PaIDI* stem	0.007
Expression	*PaIDI* needle × *PaIDI* roots	0.286
Expression	*PcIDI* leaf × *PcIDI* stem	<0.001
Expression	*PcIDI* roots × *PcIDI* stem	0.121
Expression	*PcIDI* leaf × *PcIDI* roots	0.005
Expression Ctr	*PaIDI*	1.0
Expression +MJ	*PaIDI* needle × Ctr	<0.001
Expression +MJ	*PaIDI* stem × Ctr	0.003
Expression +MJ	*PaIDI* roots × Ctr	<0.001
Expression Ctr	*PcIDI*	1.0
Expression + JA	*PcIDI* × Ctr	0.187
Expression + Cp	*PcIDI* × Ctr	0.017

Supplementary Table S8. *p*-values for Supplementary Fig. S3A

		Student's t-test
**Interaction**	**Sample**	***p*-value**
Mg^{2+}× Zn^{2+}	*Pc*IDI activity	<0.001
Zn^{2+} × Mg^{2+}+ Zn^{2+}	*Pc*IDI activity	<0.001
Mg^{2+}× Mg^{2+}+ Zn^{2+}	*Pc*IDI activity	0.783

Supplementary Table S9. *p*-values for Supplementary Fig. S5

		**ANOVA**
**Interaction**	**Sample**	***p*-value**
β-sitosterol	VC × RNAi-*PcIDI* - #2	0.9864
β-sitosterol	VC × RNAi-*PcIDI* - #3	0.8240
β-sitosterol	VC × RNAi-*PcIDI* - #5	0.9882

Supplementary Table S10. *p*-values for Supplementary Fig. S7

		**Student's t-test**
**Interaction**	**Sample**	***p*-value**
Flux	VC × RNAi-*PcIDI* - #2	0.35
Flux	VC × RNAi-*PcIDI* - #3	0.08
Flux	VC × RNAi-*PcIDI* - #5	0.44
Flux	VC × RNAi-*PcIDI* - #8	0.27

Supplementary Table S11. *p*-values for Supplementary Fig. S8

		**ANOVA**
**Interaction**	**Sample**	***p*-value**
Expression *PcHDR*1 / + IDP	day 7 10 uM × mock	0.735
Expression *PcHDR*1 / + IDP	day 7 30 uM × mock	0.932
Expression *PcHDR*1 / + IDP	day 14 10 uM × mock	0.856
Expression *PcHDR*1 / + IDP	day 14 30 uM × mock	0.988
Expression *PcHDR*1 / + IDP	day 21 10 uM × mock	0.546
Expression *PcHDR*1 / + IDP	day 21 30 uM × mock	0.694
Expression *PcHDR*2 / + IDP	day 7 10 uM × mock	0.135
Expression *PcHDR*2 / + IDP	day 7 30 uM × mock	0.072
Expression *PcHDR*2 / + IDP	day 14 10 uM × mock	0.856
Expression *PcHDR*2 / + IDP	day 14 30 uM × mock	0.988
Expression *PcHDR*2 / + IDP	day 21 10 uM × mock	0.346
Expression *PcHDR*2 / + IDP	day 21 30 uM × mock	0.594
Expression *PcIDI* / + IDP	day 7 10 uM × mock	0.175
Expression *PcIDI* / + IDP	day 7 30 uM × mock	0.779
Expression *PcIDI* / + IDP	day 14 10 uM × mock	0.105
Expression *PcIDI* / + IDP	day 14 30 uM × mock	0.279
Expression *PcIDI* / + IDP	day 21 10 uM × mock	0.975
Expression *PcIDI* / + IDP	day 21 30 uM × mock	0.910
Expression *PcHDR*1 / + Isoprenol	day 7 10 uM × mock	0.063
Expression *PcHDR*1 / + Isoprenol	day 7 30 uM × mock	0.071
Expression *PcHDR*1 / + Isoprenol	day 14 10 uM × mock	0.103
Expression *PcHDR*1 / + Isoprenol	day 14 30 uM × mock	0.275
Expression *PcHDR*1 / + Isoprenol	day 21 10 uM × mock	0.071
Expression *PcHDR*1 / + Isoprenol	day 21 30 uM × mock	0.072
Expression *PcHDR*2 / + Isoprenol	day 7 10 uM × mock	0.140
Expression *PcHDR*2 / + Isoprenol	day 7 30 uM × mock	0.735
Expression *PcHDR*2 / + Isoprenol	day 14 10 uM × mock	0.088
Expression *PcHDR*2 / + Isoprenol	day 14 30 uM × mock	0.129
Expression *PcHDR*2 / + Isoprenol	day 21 10 uM × mock	0.822
Expression *PcHDR*2 / + Isoprenol	day 21 30 uM × mock	0.830
Expression *PcIDI* / + Isoprenol	day 7 10 uM × mock	0.802
Expression *PcIDI* / + Isoprenol	day 7 30 uM × mock	0.914
Expression *PcIDI* / + Isoprenol	day 14 10 uM × mock	0.101
Expression *PcIDI* / + Isoprenol	day 14 30 uM × mock	0.256
Expression *PcIDI* / + Isoprenol	day 21 10 uM × mock	0.944
Expression *PcIDI* / + Isoprenol	day 21 30 uM × mock	0.903
